# A possible universal role for mRNA secondary structure in bacterial translation revealed using a synthetic operon

**DOI:** 10.1038/s41467-020-18577-4

**Published:** 2020-09-24

**Authors:** Yonatan Chemla, Michael Peeri, Mathias Luidor Heltberg, Jerry Eichler, Mogens Høgh Jensen, Tamir Tuller, Lital Alfonta

**Affiliations:** 1 Department of Chemistry, Ben-Gurion University of the Negev, Beer-Sheva, 8410501 Israel; 2 Department of Life Sciences, Ben-Gurion University of the Negev, Beer-Sheva, 8410501 Israel; 3 Ilse Katz Institute for Nanoscale Science and Technology, Ben-Gurion University of the Negev, Beer-Sheva, 8410501 Israel; 4 Department of Biomedical Engineering, The Iby and Aladar Fleischman Faculty of Engineering and The Sagol School of Neuroscience, Tel Aviv University, Tel Aviv, 6997801 Israel; 5 Niels Bohr Institute, University of Copenhagen, Blegdamsvej 17, 2100 Copenhagen, Denmark

**Keywords:** Synthetic biology, Sequence annotation, Ribosome

## Abstract

In bacteria, translation re-initiation is crucial for synthesizing proteins encoded by genes that are organized into operons. The mechanisms regulating translation re-initiation remain, however, poorly understood. We now describe the ribosome termination structure (RTS), a conserved and stable mRNA secondary structure localized immediately downstream of stop codons, and provide experimental evidence for its role in governing re-initiation efficiency in a synthetic *Escherichia coli* operon. We further report that RTSs are abundant, being associated with 18%–65% of genes in 128 analyzed bacterial genomes representing all phyla, and are selectively depleted when translation re-initiation is advantageous yet selectively enriched so as to insulate translation when re-initiation is deleterious. Our results support a potentially universal role for the RTS in controlling translation termination-insulation and re-initiation across bacteria.

## Abstract (web summary)

The mechanisms for regulating translation re-initiation in bacteria remain poorly understood. Here, the authors screened a library of synthetic operons and identified a ribosome termination structure that modulates re-initiation efficiency and which is conserved across bacteria.

## Introduction

To initiate protein translation, a ribosome binds and assembles an initiation complex in the area of the gene start codon^1^. When monocistronic mRNA encoding a single gene is translated, spatial considerations that could interfere with ribosome binding are largely irrelevant. However, in bacteria, where a single mRNA transcript can contain several genes clustered into an operon, translation initiation must account for the space between genes. Specifically, how does translation initiation of a downstream operon gene occur without interference from the translating ribosome of the upstream gene? Despite our considerable understanding of protein translation in bacteria, this largely remains an unanswered question. Indeed, the mechanisms which control translation initiation in operons remain a matter of debate.

In bacterial operons, the intergenic distance between most of the neighboring cistrons is shorter than 25–30 nucleotides^2,3^. This distance is too small to simultaneously accommodate one ribosome terminating on the stop codon of the proximal gene and a second ribosome initiating de novo translation on the start codon of the distal gene^3^. Translation re-initiation, a scenario whereby the terminating proximal-ribosome does not dissociate from the mRNA after termination and instead re-initiates translation on the neighboring distal cistron, alleviates this problem. Presently, the mechanisms regulating translation re-initiation are not well understood^3–5^. Specifically, regulators that determine whether a ribosome dissociates from the mRNA or remains bound to re-initiate translation have yet to be discovered. We thus considered whether mRNA secondary structure could serve this role, given how mRNA structure can affect translation at the de novo initiation^6,7^ and elongation^8,9^ steps, and can also affect translational coupling between two neighboring genes on the same operon^5,10,11^.

Using *Escherichia coli* transformed with a synthetic operon as a model system, we discover a stable mRNA secondary structure found near the stop codon, termed the ribosome termination structure (RTS), that controls the efficiency of translation re-initiation. We further report, on the basis of large-scale computational analysis, that such structures are abundant throughout bacteria. Finally, we show that RTSs are positively selected to insulate translation when re-initiation-avoidance is beneficial, yet are depleted where re-initiation could prove useful, principally in operon-clustered genes.

## Results

### mRNA structure drives distal gene expression in a synthetic operon

**Fig. 1:**
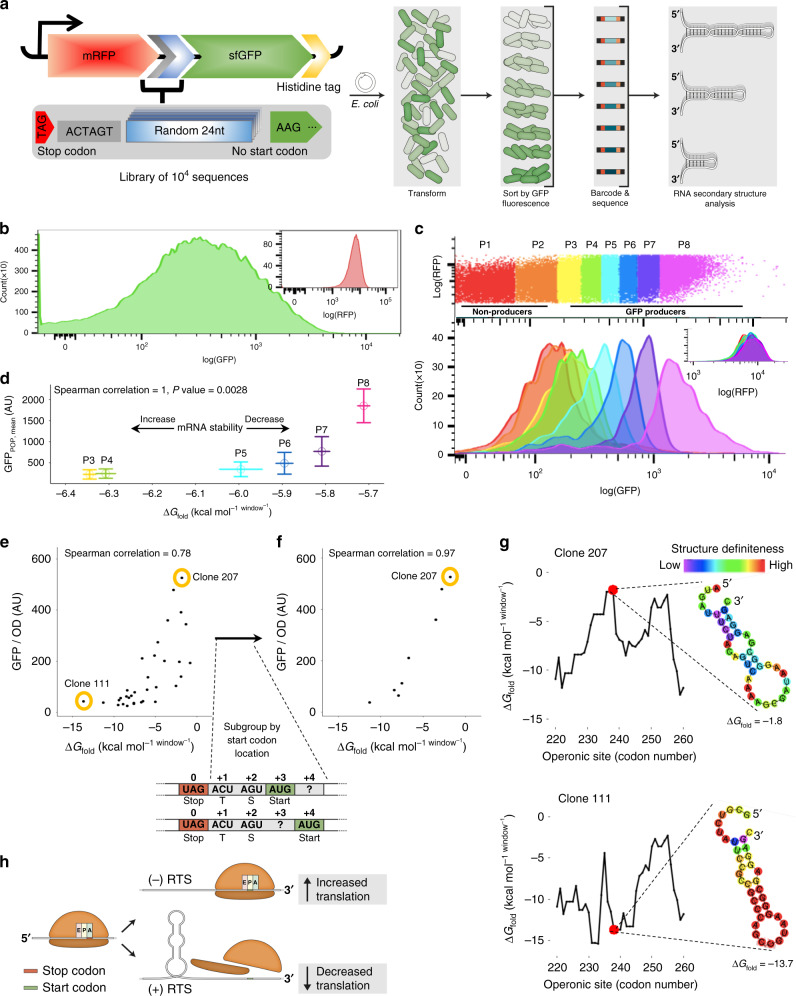
mRNA secondary structure (ΔG_fold_) controls distal operon gene expression. **a** Synthetic operon design and the FACS scheme employed. **b** GFP and RFP fluorescence of 10^5^ cells. **c** Sorting of 10^6^ cells into color-coded bins with constant RFP and variable GFP levels (top); GFP distribution in 3000 cells from each bin after sorting (bottom). **d** Correlation between the population mean GFP expression levels and the weighted mean of ΔG_fold_ of 3 × 10^3^ unique sequences in each bin. The *x* and *y* axes error bars represent the 99% confidence interval and relative standard deviation, respectively. Spearman correlation was performed on the weighted averages of the six bins (*n* = 6, *ρ* = 1, *p* value = 0.0028). Correlation between GFP expression and ΔG_fold_ of (**e**) all (*n* = 33) isolated variants, and (**f**) a subset (*n* = 8) presenting an AUG start codon at position +3 or +4. **g** ΔG_fold_ landscape around the stop codon and the mRNA secondary structure presented in the first window outside the stop codon-occupying ribosome footprint of two selected clones (111, 207). The red dot represents the *RFP* stop codon. Secondary mRNA structures of all clones are available in Supplementary data file 3. **h** Schematic representation of the role of the RTS in distal operon gene translation (ribosomes are not drawn to scale).

To test the relation between mRNA secondary structure and translation re-initiation, a library of operons based on the pRXG plasmid^12^ was assembled (Fig. 1a). These synthetic operons comprise a proximal gene encoding red fluorescent protein (RFP) and a distal gene encoding polyhistidine-tagged green fluorescent protein (GFP), separated by a stretch of 24 random nucleotides in the inter-cistronic region, downstream of the *RFP* stop codon. The library was transformed into *Escherichia coli* MG1655 cells and sorted according to GFP expression levels into eight bins spanning three orders of magnitude (Fig. 1b), using flow cytometry (Fig. 1c). Each bin was barcoded, sequenced, and the weighted Gibbs free energy average of mRNA secondary structure (ΔG_fold_) in the variable sequence region in that bin was calculated.

The first two bins (P1 and P2) exhibited GFP expression levels that were not higher than those in the negative wild-type bacteria controls (Supplementary Fig. 1). As such, bins P1 and P2 were labeled as non-producing populations and not further analyzed. The results from the other bins (P3–P8), however, revealed significant correlation between observed GFP levels and the calculated mean ΔG_fold_ of the ~3 × 10^3^ unique sequences in each bin (Spearman correlation ρ = 1, *n* = 6, *p* value = 0.0028; Fig. 1d). These results illustrate the inverse correlation between expression levels of the distal gene-encoded GFP and mRNA folding stability, such that sequences with lower stability in the variable region were significantly enriched in high GFP-producing populations, and vice versa (Supplementary Fig. 1e).

Next, individual clones from each bin were sorted and sequenced. Thirty-three clones in which the variable inter-cistronic sequence encoded at least one of the six most abundant start codons for translation initiation^13^ also lacked additional in-frame stop codons and presented a unique ΔG_fold_. These clones were isolated, and their GFP expression levels were quantified (Supplementary Table 1). Upon assessing the relation between ΔG_fold_ of the variable sequence and GFP expression, clear correlation was revealed (Spearman correlation ρ = 0.78, *n* = 33, *p* value < 10^−7^; Fig. 1e). Such correlation was independent of mRNA abundance (Supplementary Fig. 2), expression of the upstream *RFP* gene (Supplementary Fig. 3), or of the location or identity of the start codon and adjacent SD sequence in the downstream *GFP* gene to which the ribosome binds^14^ (Supplementary Table 2). No significant effect on growth rate was observed among the clones. Rather, the character of the clone-specific intergenic sequence had a significant impact on GFP levels but not on growth (Supplementary Fig. 4).

In a distinct subset of eight clones where variability in the start codon was further limited to only one of the three most used *GFP*-start codons (AUG, GUG, UUG), and variability in their position was limited to only three or four codons downstream of the *RFP* stop codon, the correlation was strengthened (Spearman correlation ρ = 0.98, *n* = 8, *p* value = 4 × 10^−4^; Fig. 1f). In this subset, in which the SD sequence was identical for all clones, the GFP expression trend was confirmed at the population level using fluorescence-activated cell sorting (FACS) analysis (Supplementary Fig. 1e). The results thus showed that distal operonic GFP gene expression is negatively affected by a stable mRNA secondary structure in the region directly downstream of the stop codon of the preceding gene (Fig. 1g and Supplementary data file 3). This structure was termed the Ribosome Termination Structure (RTS), with the likelihood of RTS presence and its strength being defined by the magnitude of ΔG_fold_ (Fig. 1h).

### The RTS is conserved across bacterial genomes

**Fig. 2:**
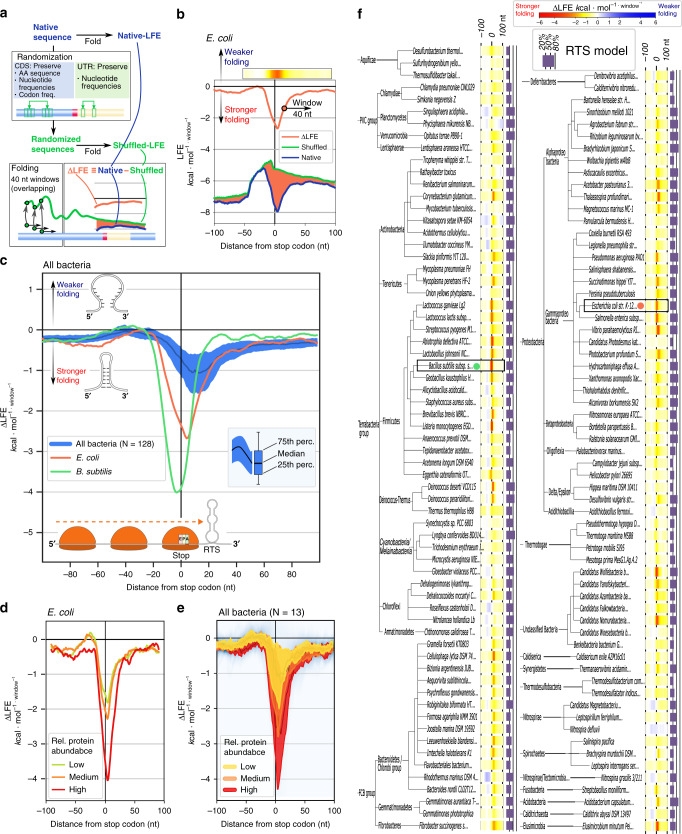
RTSs are conserved across bacterial phyla. **a** Pipeline for genome-wide RTS analysis. ∆LFE analysis reveals that on average for all genes, an RTS is present and localized downstream of stop codons across (**b**) *E. coli* (orange), **c** *B. subtilis* (green), and 128 bacterial species examined (blue). The RTS signal is more significant in genes encoding highly abundant products in (**d**) *E. coli*, and (**e**) all bacterial species for which protein abundance data is available. **f** ∆LFE heatmap depicting the 100 nucleotide-long regions around stop codons across bacteria (warm colors: stronger folding than expected; cool colors: weaker folding than expected). The purple bar, left of each species heatmap, represents the fraction of genes in which RTS was found under the RTS statistical model described in the Methods section.

To assess the generality of the RTS, mRNA secondary structure stability (∆G_fold_) was calculated in a region spanning 100 nucleotides on either side of each of the ~4200 annotated *E. coli* stop codons using a 40 nucleotide-long sliding window, allowing for the calculation of the mean ∆G_fold_ at each position in a genome-wide manner (Fig. 2a). Such analysis revealed an extreme drop in ∆G_fold_ (reflecting stronger mRNA folding), with a global minimum of $-7.94\,{\mathrm{kcal}}\,{\mathrm{mol}}^{{-1}{\mathrm{window}}^{-1}}$ centered five nucleotides downstream of the last nucleotide of a stop codon (Fig. 2b, blue line), corresponding to the expected position and magnitude of an RTS. This demonstrates that RTS-like signals are apparent throughout the *E. coli* genome.

To confirm that the RTS is directly under selection and as a control for other mRNA-stability factors, the ∆G_fold_ value of each sequence (Fig. 2b, blue line), minus the ∆G_fold_ value of a shuffled version in which nucleotide and codon content but not their order are preserved, was calculated (Fig. 2b, green line). This was repeated for each position across all *E. coli* genes, providing an average selection landscape of mRNA structure (Fig. 2b, orange line). If only nucleotide or codon content was under selection, then the difference in local folding energy (∆LFE) between native and randomized sequences should equal zero. Hence, increased ∆LFE deviation in the negative direction indicates direct selection for enhanced secondary structure stability (and vice versa). The results revealed extreme selection for stable structure directly downstream of stop codons (Fig. 2b, orange line) (Wilcoxon test, *p* value < 10^−30^), irrespective of the stop codon used (Supplementary Fig. 4). The global minimum of ∆LFE ($-2.67\,{\mathrm{kcal}}\,{\mathrm{mol}}^{{-1}{\mathrm{window}}^{-1}}$) represents strong selection for the RTS structure directly downstream of stop codons. The same signal was seen in an average of 128 other bacterial strains representing all phyla (Fig. 2c, blue line), including the evolutionary distant Gram-positive *Bacillus subtilis* (Fig. 2c, red line).

If RTS presence is indeed under selection, correlation to the level of gene expression would be expected, with genes encoding more abundant proteins being subjected to stronger selection pressure. To test this hypothesis, *E. coli* genes were grouped according to protein abundance, and the ∆LFE landscape of each was determined (Fig. 2d). Clear and significant correlation between protein abundance and ∆LFE was noted (Mann–Whitney test, *p* value < 10^−30^), demonstrating the RTS to be an adaptive trait, possibly controlling distal operon gene translation. This relation also holds true in *B. subtilis* and all 11 other bacteria for which data is available (Fig. 2e).

Lastly, RTS presence was quantified genome-wide across bacteria. This revealed that an RTS signal, defined as an mRNA structure ($\Delta{\mathrm{G}}_{\mathrm{fold}}\leq{-6}\,{\mathrm{kcal}}\,{\mathrm{mol}}^{{-1}{\mathrm{window}}^{-1}}$) directly downstream of the stop codon that is significantly more stable than the surrounding sequences (see Methods section), is present in 18%–66% of all genes, depending on the species (Fig. 2f, Supplementary Fig. 6, Supplementary data files 5–7). Genome-wide variability between species reflects a combination of selection for structural stability and the fraction of genes that are followed by an RTS.

### Translation re-initiation is controlled by RTS

**Fig. 3:**
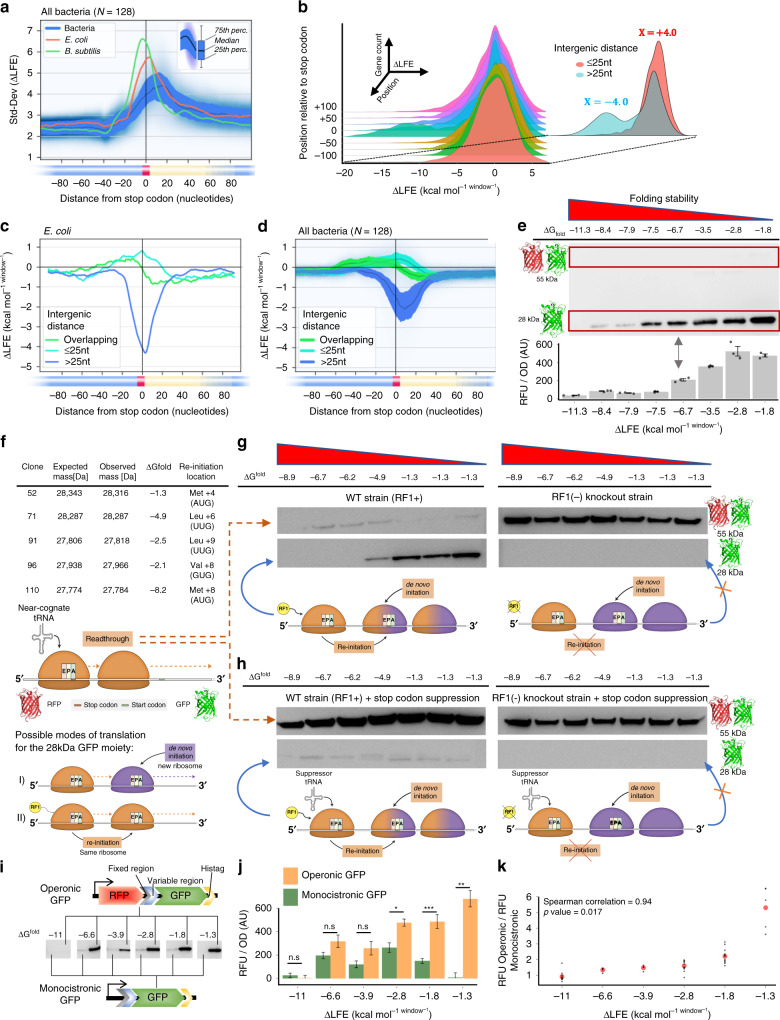
The RTS controls translation re-initiation. **a** ΔLFE standard deviation landscape around stop codons. **b** *E. coli* gene density plot (Z-axis) versus ΔLFE (X-axis) and distance from a stop codon (*Y*-axis). Different colors are used for improved visualization. Inset shows gene density at position zero. Gene pairs separated by an intergenic distance larger or smaller than 25 nucleotides are in cyan and red, respectively. Gray represents the intersection of the two groups. The RTS profile around the stop codon depends on the inter-cistronic distance before the downstream gene in (**c**) *E. coli* and (**d**) 128 bacterial species. All parameters used to calculate ΔLFE are constant across all figures, and relied on a window size of 40 nucleotides. **e** Representative anti-His-tag Western blot (top) and the mean of *n* = 3 fluorescence measurements (error bars represents standard error; bottom) of eight AUG (+3/+4) clones, with ΔG_fold_ indicated. **f** Mass spectrometry analysis of GFP from selected library clones, with the codon and location used for re-initiation indicated. Representative cropped Western blots of seven random *E. coli* clones (**g**) without or (**h**) with stop codon reassignment, each in the presence (left) or absence (right) of RF1. **i** Genetic constructs of operonic and monocistronic GFP. Each anti-His-tag Western blot represents a comparison, normalized to OD, between the two constructs for each of six tested clones. **j** The mean fluorescence measurements comparing the two constructs. Error bars represent standard deviation. Significance was determined by Welch two-sample *t*-tests (from left to right; df = 22.0, *p* = 0.4164; df = 4.5, *p* = 0.1091; df = 6.3, *p* value = 0.0854; df = 20.9, *p* value = 0.0397; df = 16.3, *p* value = 0.00061; df = 4.3, *p* value = 0.0067). **k** Spearman correlation (*n* = 6, ρ = 0.94, *p* value = 0.017), between the ratio of operonic to monocistronic GFP levels and ΔG_fold_ of each clone. Uncropped Western blots are available (Fig. S9). Ribosomes are not drawn to scale.

The precise role of the RTS was considered by examining variability in ΔLFE, distinguishing between genes followed by an RTS or not. Such analysis showed the standard deviation of ΔLFE to spike in the vicinity of the stop codon (Fig. 3a), yielding a bi-modal pattern of gene distribution only around the stop codon (Fig. 3b). The parameter best-defining the two groups of gene distribution is the inter-cistronic distance separating neighboring genes (Fig. 3b, inset). *E. coli* gene pairs separated by shorter distances (<25 nucleotides, *N* = 1537) were significantly depleted of RTSs (${\mathrm{mean}}\,\,\Delta{\mathrm{LFE}}=+{0.4}\,{\mathrm{kcal}}\,{\mathrm{mol}}^{{-1}{\mathrm{window}}^{-1}}$, Wilcoxon test, *p* value = 5 × 10^−19^); for further-separated neighboring genes (≥25 nucleotides, *N* = 2,581), RTSs were significantly enriched (${\mathrm{mean}}\,\,\Delta{\mathrm{LFE}}={-4.0}\,{\mathrm{kcal}}\,{\mathrm{mol}}^{{-1}{\mathrm{window}}^{-1}}$, Wilcoxon test, *p* value < 10^−30^).

When the ΔLFE landscape around the stop codon between gene pairs in each group was charted (Fig. 3c), RTS depletion was noted when the intergenic distance is short, or when the two consecutive cistrons overlap. Conversely, when the intergenic distance exceeds 25 nucleotides, an RTS is present (Mann–Whitney, *p* value < 10^−30^). This trend is conserved in 128 bacterial species analyzed (Fig. 3d). Considering that ~25 nucleotides are the intergenic distance below which translation re-initiation is considered to be advantageous over de novo initiation^3^, and the above-identified correlation between RTS presence and expression of the distal operonic *GFP* gene (Fig. 1), the RTS can be linked to translation re-initiation. We thus propose that RTS enrichment in the ≥25 nucleotides group and depletion from the <25 nucleotides group reflects how RTS presence serves to inhibit translation re-initiation when it is not advantageous, while its absence enables this event.

Translation of the distal partner of any operon-based gene pair can be realized by de novo initiation, translation re-initiation, or stop codon read-through. Thus, discounting a link between the RTS and de novo initiation or stop codon read-through would further support a role for the RTS in translation re-initiation. Accordingly, experiments involving the synthetic operon described above (Fig. 1a) were performed, given how expression of the distal GFP gene could result from any of the above-mentioned processes.

The link between the RTS and stop codon read-through was tested by Western blot analysis of a subgroup of clones described above (Fig. 1f) expressing the RFP-GFP synthetic operon, normalized by OD_600_, using antibodies against the GFP C-terminal polyhistidine tag. The 55 kDa RFP + GFP product resulting from stop codon read-through was barely detectable, compared to the 28 kDa GFP product resulting from de novo initiation or re-initiation (Fig. 3e). The intensities of these SDS-PAGE protein bands obtained from these clones, as well as those from other randomly selected clones, were quantified by densitometry. This confirmed that correlation between the level of the 28 kDa product and ΔG_fold_ was maintained (Spearman correlation ρ = 0.80, *n* = 58, S = 6479, *p* value < 10^−13^; Supplementary Fig. 7). Lastly, exact product masses were verified by mass spectrometry to reveal the initiation codon and its location (Fig. 3f, Supplementary Fig. 7, Supplementary Table 1). These findings thus discount linkage between RTS presence and stop codon read-through.

To determine whether the RTS is linked to de novo initiation or translation re-initiation, the manner of *GFP* translation initiation was assessed using the release factor 1 (RF1)-deficient *E. coli* C321.∆*prfA* EXP strain^15^ and Western blot analysis of random clones, as above. In the absence of RF1, the ribosome cannot efficiently terminate translation at the *RFP* UAG stop codon, thereby precluding translation re-initiation, which depends on such termination. Instead, GFP expression can only be driven by read-through or de novo initiation in the mutant strain. Western blot analysis detected only the read-through RFP + GFP product (Fig. 3g, Supplementary Fig. 8). This serves as evidence that de novo initiation does not drive GFP translation. Still, the apparent lack of de novo GFP translation initiation in the deletion strain could result from physical interference of the initiation site by *RFP*-translating ribosomes and increased read-through. To discount this possibility, the *RFP* UAG stop codon in *E. coli* MG1655 was suppressed (see “Methods”" section) so as to mimic conditions of ribosomal occupancy that may occur in RF1-deficient cells. Under these conditions, isolated GFP was produced only in the *E. coli* MG1655 strain but not in RF1-depleted cells (Fig. 3h).

Next, to directly test the ability of the intergenic region to guide de novo initiation of translation, the RFP gene and its ribosome-binding site were deleted from the operons in six selected clones. In the resulting monocistronic GFP construct, only the 18 terminal nucleobases of the RFP gene, the fixed and variable intergenic regions, and the GFP gene remain downstream of the *lac* operator (Fig. 3i). The 18 terminal nucleobases of the RFP gene were not removed to mimic the exact mRNA sequence-context encountered by initiating ribosomes in all clones. GFP levels were then compared between the monocistronic and operonic constructs of each clone, using both Western blot analysis (Fig. 3i) and fluorescence measurements (Fig. 3j).

The results revealed that when strong RTSs are present, both constructs exhibit similarly low levels of GFP expression, with the ratio of expression by the two being close to one. Conversely, in clones with weak RTSs, the operonic constructs showed significantly higher levels of GFP expression, reaching levels over five-fold higher than that of the monocistronic constructs. This observation correlates well with the ΔG_fold_ of each pair of clones (Fig. 3k) (Spearman correlation ρ = 0.94, *S* = 2, *n* = 6, *p* = 0.017). Such correlation indicates that when the RTS is less stable, the difference in GFP expression between monocistronic and operonic constructs increases, as expected according to the hypothesis that a weak RTS allows for increased translation re-initiation. These results thus demonstrate how de novo initiation is not affected by the RTS in the same manner as is translation re-initiation. Moreover, they show that the monocistronic clones recruited new ribosomes for translation initiation with very low efficiency. This low efficiency confirms that a significant part of the observed GFP expression phenotype is dependent on the presence of the upstream RFP gene and, as such, is not likely a result of de novo initiation.

Given that de novo initiation does not correlate with RTS strength, does not result in efficient expression in the monocistronic clones tested, and could not be detected when *RF1* was knocked out, argue against de novo initiation as a viable mechanism to explain the dependence of operonic distal GFP expression on the RTS. As such, we conclude that translation re-initiation remains the most likely process by which the RTS controls expression of the operonic distal *GFP* gene.

### RTS is dependent on the operonic position of a gene

**Fig. 4:**
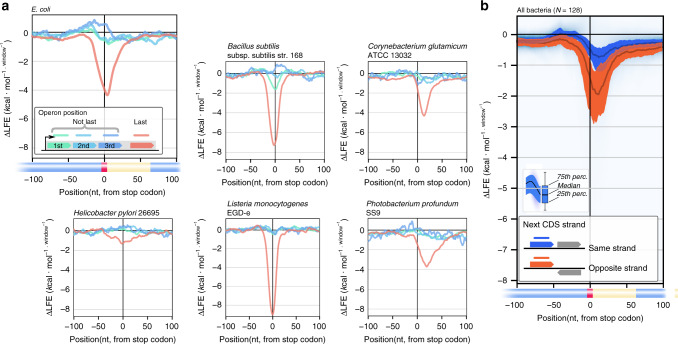
In all bacteria phyla, RTSs are enriched where re-initiation is deleterious and depleted where re-initiation is advantageous. **a** RTS presence depends on operonic position in *E. coli* and in all operon-mapped bacterial species. The blue curves represent the average ΔLFE of first and middle operon genes, while the red curve represents terminal operon genes. **b** RTS presence depends on downstream cistron directionality in 128 bacterial species.

Finally, to determine whether the translation re-initiation-controlling role assigned to the RTS can be generalized, “transcriptional unit” data^16^ cataloging the arrangement of *E. coli* genes into operons were assessed (Fig. 4a).

Such analysis revealed that downstream of all operon terminal genes, where re-initiation is deleterious, the presence of an RTS after the stop codon, possibly insulating against re-initiation, is favorable. In contrast, RTSs are depleted after the stop codon of all other operonic genes, possibly encouraging re-initiation (Mann–Whitney, *p* value < 10^−30^). These results were strengthened by observing that RTS presence after terminal operonic genes is independent of the presence or absence of start codons in the 50 nucleotide-long stretch downstream of the stop codon, while significant such dependence was seen for other operon genes (Supplementary Fig. 10). The same held true in *B. subtilis* and four other bacterial species for which experimental operon arrangement data exists (Fig. 4a).

Gene annotations in 128 bacterial species were analyzed for RTS presence as a function of neighboring gene strand directionality. Such analysis allowed for assessing operons in genomes where operons are not annotated, based on the assumption that neighboring genes on opposite DNA strands are less likely to be on the same operon than are gene pairs on the same strand. Accordingly, pairs of neighboring genes on the same strand, where re-initiation on mRNA is possible, were compared to pairs on opposite strands, where such re-initiation would be useless as the two genes cannot be transcribed as a single mRNA (Fig. 4b). As expected, RTS presence was significantly higher within gene pairs found on opposite strands, where insulation against re-initiation could help avoid translation of the 3′ UTR.

With this understanding, the source of variability between species in terms of the strength of selection for the RTS (i.e., ΔLFE values) was explored. This was performed for each of the 128 bacterial species considered, by distinguishing between gene pairs presenting intergenic distances of less than 25 nucleotides or which are on the same strand (i.e., where an RTS is less likely), and gene pairs separated by larger intergenic distances or found on opposite strands (i.e., where an RTS is more likely).

Three genome-specific parameters were examined, namely, %GC content, the number of gene pairs on opposing strands, and the average intergenic length (Supplementary Fig. 11). Although inter-species variance in RTS selection was found to be correlated to all three parameters, it is of note that the high positive correlation between ΔLFE and genomic %GC content was only seen in gene pairs where an RTS is less likely to occur (Pearson, *n* = 128, *r* = 0.546, *p* value < 10^−10^; Fig. S11). Such correlation reflects stronger selection for RTS depletion in mid-operonic genes in organisms with higher %GC content. Considering that when %GC content is high, spontaneous mRNA secondary structures are more likely to appear, we expected and indeed observed, that more substantial purifying selection is required for RTS depletion^17^.

Lastly, we explored whether RTS regions in the *E. coli* genome are enriched in any sequence motifs. Two uncharacterized motifs were identified but only in a small subset of genes, and as such, are unlikely to control re-initiation or account for RTS selection (Table S4). These results, together with the demonstrated lack of RTS linkage to transcription termination (Supplementary Figs. 2 and 12), are all consistent with the RTS playing a major role in bacterial translation re-initiation.

## Discussion

Translation re-initiation affords bacteria the ability to translate operon-clustered genes with minimal interference between terminating and initiating ribosomes. However, the capacity for translation re-initiation also carries risk. Uncontrolled re-initiated translation could evoke high fitness costs due to ribosomes devoting more time scanning for translation re-initiation sites or because of unintended translation re-initiation events. Indeed, as the ribosome can re-initiate in all possible frames and recognizes several start codons^13^ (Fig. 3f, Supplementary Table 2) even on mRNAs with alternative or lacking SD sequences^10,11^ (Supplementary Table 2), unintended translation re-initiation is of real concern. For example, if one considers the median 3′ UTR length of all *E. coli* genes (50 nucleotides; Supplementary Fig. S13d), the probability of an efficient start codon being present in the sequence, in any frame, is higher than 90% (Supplementary Fig. 13a). In agreement with this assessment, *E. coli* genome analysis reveals that ~88% of genes are followed by an efficient start codon within the 50 nucleotides downstream of their stop codon, with an average of 2.1 start codons per gene. As such, control over translation re-initiation is likely to be essential.

Here, we identified a stable mRNA secondary structure downstream of the stop codon (termed the RTS), and experimentally showed that the RTS likely controls translation re-initiation in a synthetic operon in *E. coli*. We further revealed that robust signals corresponding to RTS presence are found across the *E. coli* genome, in agreement with recently published transcriptome-wide mRNA stability data^18,19^. We also showed the RTS to be conserved across bacterial phyla, with an RTS signal peaking around a position that correlates with the edge of the mRNA stretch that is shielded by a terminating ribosome, alluding to a possible RTS-ribosome interaction. Indeed, the functional computational analyses and experiments performed here all support the RTS as acting as a translational insulator, inhibiting translation re-initiation.

This claim, however, is based on a synthetic experimental setup. Therefore, at this time, we can only speculate that the interpreted role of the RTS in genetic regulation also holds true in natural bacterial genomes. Future validation of RTS function should entail perturbation and characterization of native RTS sequences in bacterial genomes, as well as defining RTS sequences in genomes and systematically characterized these entities in synthetic reporter operons.

Our findings, moreover, do not exclude additional RTS functions. For example, we cannot exclude that in some contexts, the RTS could serve both as a Rho-independent transcription terminator and as an inhibitor of de novo initiation that can mask 3′ UTRs from unintended translation initiation. Our experimental and computational results did, however, reveal a direct link only between the RTS and translation re-initiation; no such relationship could be detected with transcription.

Further support for the role of the RTS in translation re-initiation comes from the fact that our results do not support a connection of the RTS to de novo initiation, which could not be observed with our synthetic operon in the absence of RF1, nor correlate with RTS stability (Fig. 3). At the same time, de novo initiation model predictions also did not correlate with our results (Supplementary Table 2). In addition, the expression of upstream RFP in the random library clones was not correlated with the strength of downstream GFP expression (Supplementary Fig. 3), yet significantly correlated with RTS strength. The latter would be unexpected were GFP translation de novo-initiated, as the distance between the *RFP* stop codon and the *GFP* start codon is too short (6-24 nucleotides) to allow these genes to simultaneously bear terminating and initiating ribosomes, respectively. Instead, these ribosomes must compete and be inter-dependent for binding. The expression of both genes, however, appears to be independent, as opposed to the dependency of GFP expression on the RTS.

Currently, two competing models explain re-initiation, namely the classic 30S binding model, where ribosomes dissociate from polycistronic mRNA upon gene translation termination only to immediately re-bind, as in de novo initiation, and translate the downstream cistron^14^. In this mode, one would expect translation of a distal cistron by both re-initiating and de novo initiating ribosomes, which would compete for the ribosome-binding domain. The second model is the recently demonstrated 70S scanning model, where the ribosome does not dissociate but instead scans the downstream mRNA for a re-initiation site^3,20^. Our results provide support for the latter model, as de novo initiation was not observed. Moreover, the observed existence of an RTS in terminal genes is more parsimonious when scanning-based re-initiation occurs. Although the molecular mechanism by which the RTS controls ribosomal re-initiation remains unknown, we can conjecture, given earlier reports, that it acts as an energy barrier for the scanning ribosome, which unlike the actively elongating ribosome, does not possess an energy source^3,20,21^.

In summary, the discovery of the ribosome termination structure, a possible translation re-initiation insulator, raises new questions on the function and evolution of operons and could lead to exploitation of this remarkably conserved structural moiety for better control over genetic design.

## Methods

### Strains and plasmids

The bacterial strains used in this study were *E. coli* K-12 MG1655 (Yale stock CGSC#6300) and C321.∆*prf*A EXP^15^ (Addgene #48998). For stop codon suppression by genetic code expansion, experimental strains were transformed with a pEVOL plasmid harboring the *Methanosarcina mazei* (*Mm*) orthogonal pair of *Mm*-PylRS/*Mm*-tRNA_CUA_ (Pyl-OTS)^22,23^. The synthetic operon plasmid was adapted from the pRXG dual reporter plasmid^12^ (Addgene Plasmid #113643), and the random sequence was inserted using random primer amplification followed by Gibson assembly. For this assembly, appropriate forward [TGGCTCCGCTGCTGGTTCTGGCGAATAGACTAGTNNNNNNNN NNNNNNNNNNNNNNNNAAGGGCGAGGAGCTCTTTACTG] and reverse [GGAGTCCAAGC TCAGCTAATTAAGCTTGGCTGCAGGTCGACCCGGGGTACCGAGC] primers were used. Expression of the synthetic operon was controlled by the *lac* operator so as not to affect bacterial fitness, given the variability of the random sequence, which is only expressed when IPTG (1 mM) is added to the growth media. To control for known stop codon context effects^24^, the first six nucleotides in this variable region (ACUAGU) were fixed. After assembly, the library was transformed into *E. coli* DH5α, where library complexity was measured as ~10^4^ by counting colony-forming units. The plasmid library was then purified using a Miniprep kit [Promega] and transformed into the *E. coli* MG1655 and C321 strains mentioned above. All *E. coli* MG1655 clones were subjected to FACS [FACSAria III, BD Biosciences]. In addition, individual clones were isolated using agar plating, and their plasmids were purified and sequenced (Supplementary Table 2). Each variable sequence that did not present an additional stop codon in the variable region was named pRXNG and given a running number name (i.e., pRXNG 60 is clone #60) and its RFP and GFP expression levels were measured. Deletion of the RFP gene for the experiments detailed in Fig. 3i, j was achieved by Gibson assembly using the following primers, forward: [ATAACAATTTCACACAGAAACAGAAGCTGGTTCTGGCGAATAGACTAG], reverse: [TTCTGTTTCTGTGTGAAATTGTTATCCG].

### Fluorescence-activated cell sorting

Bacterial cells were grown overnight induced with 1 mM IPTG, washed with PBS, and sorted by FACS [FACSAria III, BD Biosciences]. The entire cell population was sorted into eight bins based on constant mRFP1 fluorescence and varying Superfolder GFP (sfGFP) fluorescence, thereby normalizing sfGFP levels to those of mRFP1. Each bin, generated using an 85-micron nozzle at minimal flow, accounted for ~12.5% of the entire population. The 8 sorted bins were re-run to map sorting accuracy, which was found to be high (~90% of cells were distributed within 3 bins around any selected bin). Controls consisted of bacterial cells that did not contain the synthetic operon plasmid. Analysis was performed, and figures were created using FlowJo software version 10.6.1.

The gating strategy was as follows: The preliminary FSC-A/SSC-A gates were 630–17,000 and 60–3000, respectively, the SSC-W/SSC-H gates were 0–110,000 and 450–45,000, respectively, and the FSC-W/FSC-H gates were 12,000–62,000 and 200–4000, respectively. Cells that expressed RFP, which served as the positive and normalizing control with levels between 3500 and 15,000, were further gated. Next, the resulting population (49.7% of the total population) was gated into 8 ~equal groups divided and defined by GFP expression. Each group was intended to represent ~12.5% of the parent population. Statistical parameters used are detailed in Supplementary Table 3.

### Library construction, next-generation sequencing, and data analysis

Isolated bacteria from each bin were transferred into LB media, grown for 8 h at 37^o^C, harvested, and subjected to plasmid extraction using a Miniprep kit [Promega]. Library construction for Illumina MiSeq next-generation sequencing was performed according to the Illumina metagenomic protocol^25^, with adapter and primer sequences detailed in Supplementary Table 1. In each bin, a 118 bp synthetic operon amplicon, which includes the variable region, was PCR-amplified. After two rounds of amplification, the Illumina primer sequence, unique hepta-nucleotide indexes, and adapters were added to each amplicon library. The libraries were then sequenced using the Illumina MiSeq V2 reagent (300 cycles) kit. The resulting sequencing data were processed and parsed with the DADA2 package for R^26^. All identical sequence reads in each bin were aggregated, and the 10,000 most abundant sequences of each bin were obtained (Supplementary data file 1). In the eight bins, the minimal sequence depth was 2–10 reads. From the 10,000 unique sequences of each bin, all sequences that contained an additional stop codon in the variable region were removed, and the remaining sequences were filtered to include only sequences with one of the three efficient start codons (ATG, GTG, TTG)^13^ in any in-frame position of the variable region. This process resulted in *N* = 2580–2694 unique sequences in each bin (Supplementary data file 2). Notably, these unique sequences overlapped between bins, although their frequency in each bin varied. The weighted mean of ΔG_fold_ and the 99% confidence interval were calculated for each bin (see computational method for calculation), and the statistical significance comparing each pair of consecutive bins was determined using a two-tail Wilcoxon rank test.

### RFP and GFP expression from the dual reporter of the random library

Measurements from triplicate bacterial cultures grown in a 96-well plate [Thermo Scientific] covered with Breathe-Easy seals [Diversified Biotech] were recorded overnight using a 37^o^C incubated plate reader [Tecan]. RFP (excitation: 584 nm; emission: 607 nm) and sfGFP (excitation: 488 nm; emission: 507 nm) expression levels and OD_600_ were measured every 15 min. The values presented the plateau value of each clone, which was measured in at least three experimental repeats (n≥3). We reasoned a priori that normalizing fluorescence levels to OD was appropriate, as over-expression of the reporters between clones could have led to changes in total protein amounts among clones. Normalizing to OD, as a proxy for cell number per well, was more relevant for comparing GFP expression and for comparison between the Western blots and fluorescent measurement, which were also normalized to OD.

### Western blots

Bacterial cultures were normalized to the same OD_600_, after which 10 μL aliquots were mixed with 10 μL MOPS buffer and 5 μL SDS buffer and incubated for 10 min at 70^o^C. Samples were separated in 4–20% SDS gels [Genscript] and transferred to a PVDF membrane [Bio-Rad] using an E-blot protein transfer apparatus [Genscript]. After transfer, anti-His tag antibodies [his-probe (H-3) antibodies, Santa Cruz Biotechnology, sc-8036, Lot #B2317] were used to probe the transferred proteins at a dilution ratio of 1:2000. Antibody binding was visualized using an ImageQuant LAS 4000 imager [Fujifilm]. Densitometry analysis was performed using the gel tool in ImageJ V1.52a software.

### Stop codon suppression by genetic code expansion

Genetic code expansion by stop codon suppression was introduced to suppress the UAG stop codon in *E. coli* MG1655, where the unnatural amino acid N-propargyl-l-lysine (1 mM final concentration in culture) was incorporated in response to the UAG stop codon at the end of the RFP gene using the *Mm* pyrrolysine ${\mathrm{tRNA}}_{\mathrm{CUA}}^{\mathrm{pyl}}$ and pyrrolysyl-tRNA synthetase orthogonal pair^27^, expressed from the pEVOL plasmid^22,23^. Induction of PylRS was performed by adding 0.5% L-arabinose [Sigma-Aldrich] to the growth medium.

### Quantitative PCR

Quantitative PCR was performed according to MIQE guidelines^28^. *E. coli* MG1655 cells were transformed with the pRXNG clones and grown to logarithmic phase (OD_600_ of 0.4–0.5), harvested, and extracted with a GeneJET RNA purification kit [Thermo Scientific] for total RNA extraction, yielding 50 μL of RNA with a concentration of ~400 ng μL^−1^ and of high purify (A_260_/A_280_ = 2.1). This step was followed by DNase (RNase free) [Thermo Scientific] digestion using the kit protocol and guidelines. RNA was immediately reverse-transcribed into cDNA with an iScript cDNA Synthesis kit [Biorad], under kit guidelines with 1 μg RNA. Real-time PCR was performed using a KAPA SYBR FAST qPCR reagent [Sigma] in a CFX qPCR instrument [Bio Rad], with duplicates of 10 μL reactions containing 1.2 μL of cDNA in each well of a qPCR 384 well-plate [Bio Rad]. The thermocycler parameters were set to 94^0^C for 2 min, 40 cycles of 94^0^C for 15 sec, 59^0^C for 25 sec, and 72^0^C 30 sec. Two synthetic operon sample amplicons were targeted: (1) an RFP target, upstream of the variable region, between positions 394–528 with a length of 135 bases; forward primer: [GACGGTCCGGTTATGCAGAA], reverse primer: [TTCAGCGTCGTAGTGACCAC]; (2) a GFP target, downstream of the variable region, between positions 873–1008 with a length of 136 bases; forward primer: [CAAGCTCCCAGTACCATGGC], reverse primer: [GCGCTCTTGTACATAGCCCT]. In addition, a normalizing gene (16 S rRNA) was used with primers 1369F-[CGGTGAATACGTTCYCGG] and 1492R-[GGTTACCTTGTTACGACTT]. Both melt curves and agarose gel electrophoresis were used to confirm primer specificity. For all primers, only one amplicon of the correct size was detected. Sample primer pair calibration curves presented r^2^ values of 0.991 and 0.998 for primers 1 and 2, respectively, with a dynamic range between Cq 3 and 18, while the LOD was Cq 14.18. The normalizing gene primer calibration curve presented an r^2^ value of 0.996 with a dynamic range between Cq 15 and Cq 23, while the LOD was Cq 14.56. Data analysis was manually performed using Bio-Rad CFX Manager V3.1 software.

### Protein purification and mass spectrometry analysis

Proteins were fused to a 6xHis tag and purified by nickel resin affinity chromatography. Purified protein samples were analyzed by LC-MS [Finnigan Surveyor/LCQ Fleet, Thermo Scientific].

### Calculation of ΔG_fold_ for synthetic operon clones

All calculations were made using the Vienna package^29^ (ViennaRNA version 2.4.9, default settings), with the extracted mRNA sequence window upon which ΔG_fold_ calculations were made for each clone obeying the two following constraints: First, the start of the window was +9 nucleotides from the first nucleotide of the UAG stop codon. This was done to simulate mRNA secondary structure, which exists outside the ribosomal entry tunnel. Second, the window size used was experimentally determined in the limited range between 30–50 nucleotides (length of the random region of interest = 24 nucleotides). However, our analysis was not sensitive to this parameter, and the results were robust in all window sizes across the entire reasonable range. Optimal correlation between ΔG_fold_ and GFP expression was found with a window size of 37 nucleotides. As such, this window size was used to generate the results presented.

### Simulation of theoretical ΔG_fold_ of random library clones

Each set of 10^6^ random sequences was sampled from a population of uniform nucleotide distribution and filtered as follows. (i) 37nt sample: Include random sequences of length 37nt containing in-frame one of the start codons (AUG, GUG, UUG) and not containing one of the stop codons (UGA, UAG, UAA). (ii) 24 + 13 sample: this sample is mimicking the sequences of the random library used herein. It includes random sequences of length 24nt containing in-frame one of the start codons (AUG, GUG, UUG) and not containing one of the stop codons (UGA, UAG, UAA), and concatenated with the suffix [AAGGGCGAGGAGC] (giving a total length of 37nt). (iii) Unconstrained sample: Include random sequences of length 37nt.

### Species selection

Species were chosen for taxonomic diversity and overlapped with public datasets (*N* = 183), with emphasis on bacteria (*N* = 128) and archaea (*N* = 49; presented in Fig. S6). Genomic sequences and annotations were obtained from the Ensemble database^30^.

### ΔLFE (folding bias) calculations

To estimate the tendency of short-range interactions within the mRNA strand to form stable secondary structures, i.e., Local Fold Energy (LFE), sequences were broken into 40 nucleotide-long windows, and the minimum folding energy was calculated using RNAfold from the Vienna package^29^ (using default settings). To identify regions where strong or weak secondary structure may be functional, rather than reflecting a side effect of selection acting on the amino acid sequence, or nucleotide or codon composition (see Randomization, below), the influence of these factors was controlled by comparing LFE of the native sequence to that of a set of randomized sequences maintaining these factors. The difference between the LFE of the native and randomized sequences is denoted as ΔLFE or local folding bias. If only the amino acid sequence, nucleotide composition, and codon composition are under selection at a given position, one expects ΔLFE to be close to 0. Any statistically significant deviation from this value indicates that additional factors maintained under selection are needed to explain the measured native LFE value.

Since this study focused on mRNA, only those regions surrounding protein-coding genes were included; genes shorter than 40 nucleotides were excluded. Genes with a length that is not a multiple of 3, those containing an internal stop codon or where the last codon is not a stop codon were also excluded. To identify features related to translation termination, ΔLFE for all included genes from a given species was averaged at each position relative to the stop codon. All *E. coli* gene results are available in Supplementary data file 4, and results for the 128 species analyzed are available in Supplementary data files 5a–c. Table parameter annotations are detailed in the Supplementary information.

### Randomization

The randomized sequences were sampled from the distribution representing the null hypothesis, namely that only the amino acid sequence, nucleotide, and codon composition (see below) are under selection at a given position in the coding sequence. To produce random sequences maintaining these properties, synonymous codons within each coding sequence were randomly permutated, and the nucleotides of each UTR were randomly permutated. Regions overlapping multiple coding sequences were maintained without permutations. Codons containing one or more ambiguous nucleotides (N bases) were likewise maintained without permutation. Synonymous codons were identified according to the gene translation table for each species. Randomization of the non-coding UTR regions were randomized by permutating only the nucleotide composition.

### RTS model

To estimate the number of genes within each species likely to present an RTS after its stop codon, we examined each gene in all species. The RTS was defined and deemed present if three conditions were met: 1. The gene is separated from its successor by an annotated intergenic region of 25 nucleotides or more, or the next gene is on the opposite DNA strand; 2. At least five consecutive windows opening in the range of −10 to +20 nucleotides (meaning that the windows cover the region of between the −10 to +59 nucleotides, as the window size is 40, relative to the end of the stop codon), and that the ΔLFE is negative; and 3. A threshold of $\Delta{\mathrm{G}}_{\mathrm{fold}}\leq{-6}\,{\mathrm{kcal}}\,{\mathrm{mol}}^{{-1}{\mathrm{window}}^{-1}}$ must be crossed in at least one of the five or more negative ΔLFE windows. If all conditions are met, the longest consecutive stretch of windows (5 or more) would be defined as a putative RTS, and the gene will be counted as being followed by an RTS. By repeating this process for all annotated genes of a given species, the fraction of genes followed by an RTS can be calculated. All parameter values used to define an RTS in this model are preliminary, but the parameter sensitivity of the model is low, and the results are robust in large parameter space. Results for all *E. coli* genes are available in Supplementary data file 6, and file parameter annotations are available in the Supplementary information section.

### Plotting

Distributions of multiple genes or averages for multiple species are presented using statistics commonly used for boxplots, as follows. The shaded region spans the 25th and 75th percentiles, with the median plotted as a darker line. Elements outside this region are presented according to their density (blue shading in the background). Densities are shown as kernel density estimates (KDEs), computed separately at each position, using a Gaussian kernel with a bandwidth of 0.5. Plots were created using Scikit Learn (version 1.3.2)^31^ and Matplotlib (version 3.1.1)^32^. Taxonomic trees are based on NCBI taxonomy^33^ and were plotted using the ETE3 toolkit (version 3.1.1)^34^.

### Statistical analysis

All statistical analysis was performed under the guidelines of the tests described in-text. The minimal *p* value noted in the text was selected to be 10^−30^. In all cases where the precise *p* value calculated was smaller (i.e., more significant), the test-statistic score is given. To test whether ΔLFE values for a one-sample group of genes are statistically different, as compared to a reference value (e.g., for the RTS model), the Wilcoxon signed-ranks test was used on the ΔLFE (randomized ΔG-native ΔG) values for all genes (20 randomization repetitions for each gene). To test whether ΔLFE values for two-sample groups of genes are statistically different from each other, the Mann–Whitney *U* test was used on the ΔLFE (randomized ΔG-native ΔG) values for all genes (with 20 randomization repetitions for each gene). As such, the test N was 20 times the number of data points of the original sample. The *p* values and test statistics are reported for the position of the most extreme test-statistic, whereas the surrounding regions showed consistent and significant results. Detailed statistical parameters are available in Table S3.

### Code writing and computational tools throughout the computational analysis

For code writing, simulation, and analysis thereof throughout this work, the following packages were used: R package ggplot (version 3.2.1), R package data2 (version 1.14), Python (version 3.7.3), Numpy (version 1.18.1) Scikit, (version 1.3.2), Biopython (version 1.74), Pandas (version 0.25.3) Metplotlib (version 3.1.1).

### Reporting summary

Further information on research design is available in the Nature Research Reporting Summary linked to this article.

## Supplementary information

Description of Additional Supplementary Files

supplementary information

Data set 1

Data set 2

Data set 3

data set 4

data set 5

data set 6

data set 7

data set 8

data set 9

data set 10

Reporting Summary

## Data Availability

Experimentally determined operonic positions were obtained from ODB4^35^. Protein abundance data were obtained from PaxDb^36^. Experimentally determined 3′-UTR lengths were obtained from regulondb^37^. Termination type data for E. coli genes were obtained from WebGesTer^38^. Source data are provided with this paper.

## Code availability

All custom codes used to generate the results described in this article are available at the GitHub public repository [https://github.com/michaelpeeri/rnafold-rts-public].

## References

[1] Simonetti A (2008). Structure of the 30S translation initiation complex. Nature.

[2] Huber M (2019). Translational coupling via termination-reinitiation in archaea and bacteria. Nat. Commun.

[3] Yamamoto H (2016). 70S-scanning initiation is a novel and frequent initiation mode of ribosomal translation in bacteria. Proc. Natl Acad. Sci..

[4] Gunišová S, Hronová V, Mohammad MP, Hinnebusch AG, Valášek LS (2018). Please do not recycle! Translation reinitiation in microbes and higher eukaryotes. FEMS Microbiol. Rev..

[5] Levin-Karp A (2013). Quantifying translational coupling in E. coli synthetic operons using RBS modulation and fluorescent reporters. ACS Synth. Biol..

[6] Kudla G, Murray AW, Tollervey D, Plotkin JB (2009). Coding-sequence determinants of gene expression in Escherichia coli. Science.

[7] Cambray G, Guimaraes JC, Arkin AP (2018). Evaluation of 244,000 synthetic sequences reveals design principles to optimize translation in Escherichia coli. Nat. Biotechnol..

[8] Tuller T (2011). Composite effects of gene determinants on the translation speed and density of ribosomes. Genome Biol..

[9] Gorochowski TE, Ignatova Z, Bovenberg RAL, Roubos JA (2015). Trade-offs between tRNA abundance and mRNA secondary structure support smoothing of translation elongation rate. Nucleic Acids Res..

[10] Stirchak EP, Summerton JE, Weller DD (1989). Translational reinitiation in the presence and absence of a Shine and Dalgarno sequence. Nucleic Acids Res..

[11] Osterman IA, Evfratov SA, Sergiev PV, Dontsova OA (2013). Comparison of mRNA features affecting translation initiation and reinitiation. Nucleic Acids Res..

[12] Monk JW (2017). Rapid and inexpensive evaluation of nonstandard amino acid incorporation in Escherichia coli. ACS Synth. Biol..

[13] Hecht A (2017). Measurements of translation initiation from all 64 codons in E. coli. Nucleic Acids Res..

[14] Kozak M (1999). Initiation of translation in prokaryotes and eukaryotes. Gene.

[15] Lajoie MJ (2013). Genomically recoded organisms expand biological functions. Science.

[16] Gama-Castro S (2016). RegulonDB version 9.0: High-level integration of gene regulation, coexpression, motif clustering and beyond. Nucleic Acids Res..

[17] Peeri M, Tuller T (2020). High-resolution modeling of the selection on local mRNA folding strength in coding sequences across the tree of life. Genome Biol..

[18] Del Campo C, Bartholomäus A, Fedyunin I, Ignatova Z (2015). Secondary structure across the bacterial transcriptome reveals versatile roles in mRNA regulation and function. PLoS Genet..

[19] Burkhardt DH (2017). Operon mRNAs are organized into ORF-centric structures that predict translation efficiency. Elife.

[20] Adhin MR, J. Van D (1990). Scanning model for translational reinitiation in eubacteria. J. Mol. Biol..

[21] Osterman IA, Evfratov SA, Sergiev PV, Dontsova OA (2012). Comparison of mRNA features affecting translation initiation and reinitiation. Nucleic Acids Res..

[22] Young TS, Ahmad I, Yin JA, Schultz PG (2010). An enhanced system for unnatural amino acid mutagenesis in E. coli. J. Mol. Biol..

[23] Chemla Y, Ozer E, Schlesinger O, Noireaux V, Alfonta L (2015). Genetically expanded cell-free protein synthesis using endogenous pyrrolysyl orthogonal translation system. Biotechnol. Bioeng..

[24] Chemla Y, Ozer E, Algov I, Alfonta L (2018). Context effects of genetic code expansion by stop codon suppression. Curr. Opin. Chem. Biol..

[25] Illumina. 16s metagenomic sequencing library preparation. Preparing 16S Ribosomal RNA gene amplicons for the Illumina MiSeq system. 1–28 (2013).

[26] Callahan BJ (2016). DADA2: high-resolution sample inference from Illumina amplicon data. Nat. Methods.

[27] Srinivasan G, James CM, Krzycki JA (2002). Pyrrolysine encoded by UAG in Archaea: charging of a UAG-decoding specialized tRNA. Science.

[28] Bustin SA (2009). The MIQE guidelines: minimum information for publication of quantitative real-time PCR experiments. Clin. Chem..

[29] Lorenz R (2011). {ViennaRNA} package 2.0. Algorithms Mol. Biol..

[30] Cunningham F (2019). Ensembl 2019. Nucleic Acids Res..

[31] Pedregosa F (2011). Scikit-learn: machine learning in Python. J. Mach. Learn. Res..

[32] Hunter JD (2007). Matplotlib: a 2D graphics environment. Comput. Sci. Eng..

[33] Agarwala R (2018). Database resources of the national center for biotechnology Information. Nucleic Acids Res..

[34] Huerta-Cepas J, Serra F, Bork P (2016). ETE 3: reconstruction, analysis, and visualization of phylogenomic data. Mol. Biol. Evol..

[35] Okuda S, Yoshizawa AC (2011). ODB: A database for operon organizations, 2011 update. Nucleic Acids Res..

[36] Wang M (2012). PaxDb, a database of protein abundance averages across all three domains of life. Mol. Cell. Proteom..

[37] Santos-Zavaleta A (2019). RegulonDB v 10.5: tackling challenges to unify classic and high throughput knowledge of gene regulation in E. coli K-12. Nucleic Acids Res..

[38] Mitra A, Kesarwani AK, Pal D, Nagaraja V (2011). WebGeSTer DB-A transcription terminator database. Nucleic Acids Res..

