# Supplementary material for: A possible universal role for mRNA secondary structure in bacterial translation revealed using a synthetic operon
Source: Nat Commun. 2020 Sep 24;11:4827. doi: 10.1038/s41467-020-18577-4 (PMC7518266; doi:10.1038/s41467-020-18577-4)
Supplement: Supplementary file 1 — Description of Additional Supplementary Files [file 41467_2020_18577_MOESM1_ESM.pdf]

Title: Supplementary Data set 1:
Description: all NGS sequences

Title: Supplementary Data set 2:
Description: final NGS dataset for Fig 1

Title: Supplementary Data set 3:
Description: mRNA secondary structure of all clones

Title: Supplementary Data set 4:
Description: E. coli figures 2-4 data

Title: Supplementary Data set 5:
Description: raw data for figure S5a

Title: Supplementary Data set 6:
Description: raw data for figure S5b

Title: Supplementary Data set 7:
Description: raw data for figure S5c

Title: Supplementary Data set 8:
Description: raw data for figure S6b

Title: Supplementary Data Set 9:
Description: Supplementary data table 1 - Library details for NGS in MiSEQ Faculty of Natural Sciences Dept. of Chemistry & Life Sciences

Title: Supplementary Data set 10:
Description: Supplementary data table 2 - Synthetic operon clone sequences (from +1 to +70 relative to stop codon)
