# Supplementary material for: A possible universal role for mRNA secondary structure in bacterial translation revealed using a synthetic operon
Source: Nat Commun. 2020 Sep 24;11:4827. doi: 10.1038/s41467-020-18577-4 (PMC7518266; doi:10.1038/s41467-020-18577-4)
Supplement: Supplementary file 2 — supplementary information [file 41467_2020_18577_MOESM2_ESM.pdf]

Supplementary Information

A possible universal role for mRNA secondary structure in bacterial translation revealed using a synthetic operon

Chemla et al.

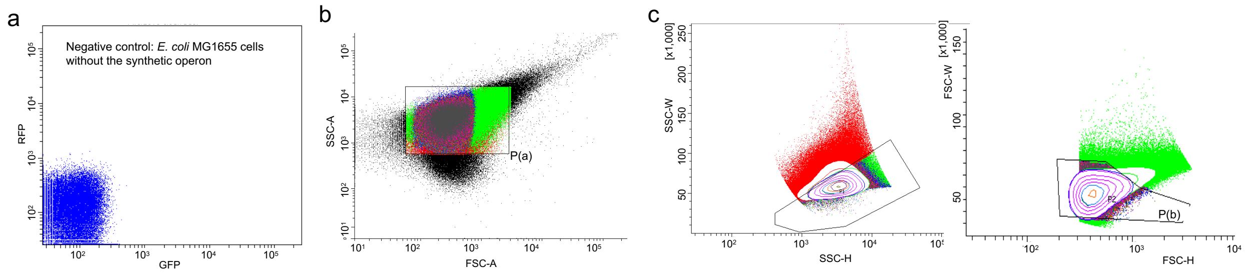

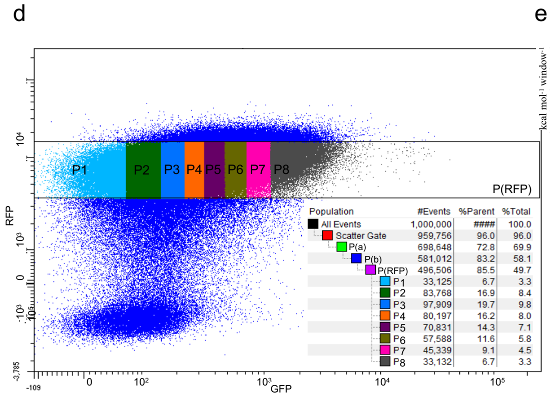

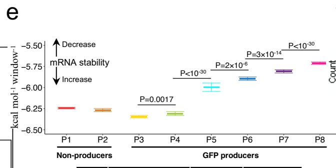

Bin	Average ΔG_{fold}	Average GFP (AU)	Average RFP (AU)
P1	-6.24	185	7,500
P2	-6.27	196	7,448
P3	-6.34	224	7,991
P4	-6.31	243	7,753
P5	-5.99	346	7,884
P6	-5.89	488	7,627
P7	-5.81	771	7,797
P8	-5.71	1,854	8,758

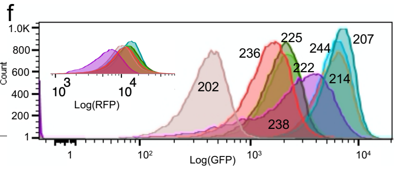

	Clone	ΔG_{fold}	Count	GFP
	202	-11.3	20,266	451
	238	-8.4	34,702	3,112
	236	-7.9	28,367	1,664
	225	-7.6	36,586	2,217
	222	-6.6	29,439	2,129
	244	-3.5	26,234	7,057
	214	-2.8	24,689	6,415
	207	-1.8	27,279	6,460

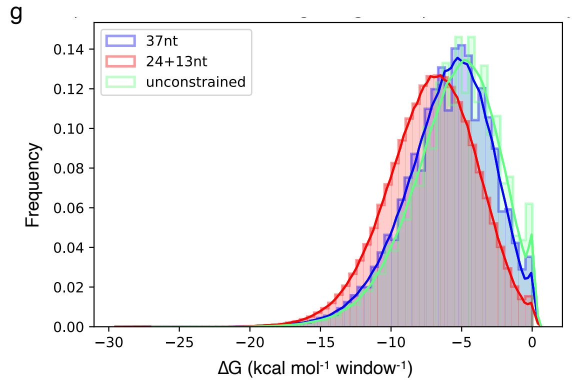

Simulation statistics

Simulation	37	24+13	Unconstrained
N	1000000	1000000	1000000
Mean	-5.987	-7.146	-5.554
Median	-5.70	-7.00	-5.30
StdDev	3.06	3.16	3.07
Min	-27.60	-28.90	-26.30
Max	0.00	0.00	0.00

Supplementary Figure 1. Flow Cytometry gating and negative control. **a**) A negative control, which consists of WT *E. coli* MG1655 **b**) First size gating **c**) Second size gating **d**) Uncropped sorting with gate and population statistics. **e**) The weighted mean of ΔG_{fold} with 99% confidence intervals of N=~3×10^3 unique sequences in each bin. Significance levels by two-sided Wilcoxon test (Table S3). Error bars represent the 99% confidence intervals. **f**) Sorting by GFP fluorescence of the eight-clone subgroup where one of the three most abundant start codons are present in position +3 or +4 from the RFP stop codon. An increase in GFP levels in each clone population negatively correlates with the increase in the negative value of ΔG_{fold} of the intergenic region between the *RFP* and *GFP* genes. **g**) Simulated RNA folding of large representative samples ($n=10^6$) from the sequence-space under constraints that were imposed on the random library (24 random followed by 13 fixed nucleotides; 24+13nt; red), under the constraints but with all 37 nucleotides randomized (37nt; blue), and unconstrained (green). The folding energies of all sample populations are gamma-distributed as expected, sample statistics are summarized in the figure table; all units are kcal mol^{-1} $window^{-1}$. The statistical values are in agreement with our experimental results, which show that all populations clustered around the constrained means, as detailed in the manuscript. If one considers that the FACS sorting and GFP expression of individual bacteria are both noisy, this simulation could well explain the central tendency of population distribution we observed in our study.

Supplementary Table 1. Characterization of individual clones sequenced from the random library
All sequences are available as tables in the data section

Clone	ΔG_{fold} [kcal/ mol/ window]	Average fluorescence RFP/OD [AU] ± SE	Average fluorescence GFP/OD [AU] ± SE	Average RFP/GFP	Best re-start codon	Start codon position*	Codon rank [1]	MS Verification
**29**	-8.9	697±254	32±4	18.5	I (AUU)	+8	6th	
**33**	-9.4	602±174	48±5	11.7	V (GUG)	+8	2nd	
**52**	-1.3	1293±269	344±12	3.8	M (AUG)	+5	1st	
**56**	-8.5	624±248	62±4	9.1	V (GUG)	+6	2nd	Yes
**57**	-6.5	923±416	43±7	19.1	L (UUG)	+8	3rd	
**62**	-6.7	474±94	39±4	12.5	L (CUG)	+9	4th	
**71**	-4.9	853±153	98±4	8.8	L (UUG)	+6	3rd	
**91**	-2.5	1155±732	103±4	13.4	L (UUG)	+9	3rd	Yes
**96**	-2.1	1161±452	197±7	5.8	V (GUG)	+8	2nd	Yes
**101**	-6.0	496±64	74±24	6.7	L (UUG)	+6	3rd	Yes
**110**	-8.2	759±228	57±4	14.8	M (AUG)	+8	1st	
**111**	-13.7	486±62	43±3	10.8	I (AUU)	+5	6th	Yes
**202**	-11.3	362±126	38±2	9.0	V (GUG)	+4	2nd	
**203**	-7.6	320±82	33±6	9.4	L (UUG)	+7	3rd	
**207**	-1.8	1236±541	526±53.5	2.1	M (AUG)	+3	1st	
**208**	-1.9	1163±664	140±19	6.5	M (AUG)	+7	1st	
**209**	-4.7	276±42	274±8	1.1	M (AUG)	+7	1st	
**212**	-5.9	287±83	137±5	2.0	M (AUG)	+3	1st	
**214**	-2.8	313±75	478±17	0.7	M (AUG)	+3	1st	
**216**	-0.8	360±78	193±17	1.9	M (AUG)	+5	1st	
**220**	-3.9	354±112	201±16	1.7	M (AUG)	+6	1st	
**222**	-6.6	333±104	211±13	1.7	M (AUG)	+3	1st	
**225**	-7.5	319±23	78±4	4.0	L (UUG)	+3	3rd	
**226**	-9.2	367±24	56±6	6.8	L (UUG)<br>M (AUG)	+5<br>+9	3rd<br>1st	
**230**	-5.0	320±29	41±6	8.2	M (AUG)	+8	1st	
**232**	-7.6	378±34	36±5	10.3	M (AUG)	+6	1st	
**233**	-8.4	398±37	25±5	18	V (GUG)	+8	2nd	
**235**	-9.5	282±13	29±5	10	L (CUG)	+5	4th	
**236**	-7.9	402±58	65±4	6.5	L (UUG)	+3	3rd	
**238**	-8.4	367±41	86±5	4.4	V (GUG)	+4	2nd	
**244**	-3.5	362±54	360±5	1	M (AUG)	+4	1st	
**245**	-5.1	411±48	222±11	1.9	M (AUG)	+7	1st	
**249**	-1.9	406±50	391±18	1.1	M (AUG)	+7	1st	

*From stop codon (distance in codons)

Supplementary Table 2. RBS calculator predictions compared to observed measurements.
Candidate ribosome binding sequences (RBS), including their Shine Dalgarno (SD) sequences, were predicted using the RBS calculator[2] that both identifies and scores possible translation initiation sites, based on the 30S binding model for *de novo* translation initiation. The *de novo* initiation predictions showed no significant correlation with the observed GFP levels (r^2=0.08), with the levels of expression observed being generally more substantial than the predictions. This strengthens the argument that the expression of the distal operon gene encoding GFP to be mainly the result of re-initiation and not *de-novo* initiation.

Clone	ΔG_{fold}	Best re-initiation start codon candidate(s)	Start codon position, relative to stop codon	Codon rank [1]	Observed translation rate ± SE [AU]	Predicted translation rate [AU] [2]	RBS binding energy ΔG_{total} [kcal/mol] [2]
**29**	-8.9	I (AUU)	+8	6th	32±4	0	NA
**33**	-9.4	V (GUG)	+8	2nd	48±5	1.34	15.16
**52**	-1.3	M (AUG)	+5	1st	344±12	90.75	5.80
**56**	-8.5	V (GUG)	+6	2nd	62±4	6.31	11.72
**57**	-6.5	L (UUG)	+8	3rd	43±7	0.49	17.40
**62**	-6.7	L (CUG)	+9	4th	39±4	0	NA
**71**	-4.9	L (UUG)	+6	3rd	98±4	13.79	9.98
**91**	-2.5	L (UUG)	+9	3rd	103±4	0.96	15.91
**96**	-2.1	V (GUG)	+8	2nd	197±7	5.76	11.92
**101**	-6.0	L (UUG)	+6	3rd	74±24	35.90	7.86
**110**	-8.2	M (AUG)	+8	1st	57±4	0.66	16.72
**111**	-13.7	I (AUU)	+5	6th	43±3	0	NA
**202**	-11.3	V (GUG)	+4	2nd	38±2	0.33	18.29
**203**	-7.6	L (UUG)	+7	3rd	33±6	2.10	14.16
**207**	-1.8	M (AUG)	+3	1st	526±53.5	16.52	9.58
**208**	-1.9	M (AUG)	+7	1st	140±19	32.12	8.11
**209**	-4.7	M (AUG)	+7	1st	274±8	479.88	2.10
**212**	-5.9	M (AUG)	+3	1st	137±5	62.34	6.63
**214**	-2.8	M (AUG)	+3	1st	478±17	11.74	10.34
**216**	-0.8	M (AUG)	+5	1st	193±17	150.90	4.67
**220**	-3.9	M (AUG)	+6	1st	201±16	1011.93	0.44
**222**	-6.6	M (AUG)	+3	1st	211±13	1.78	14.53
**225**	-7.5	L (UUG)	+3	3rd	78±4	1.61	14.76
**226**	-9.2	L (UUG)<br>M (AUG)	+5<br>+9	3rd<br>1st	56±6	0.70<br>3.31	16.59<br>13.16
**230**	-5.0	M (AUG)	+8	1st	41±6	3.72	12.90
**232**	-7.6	M (AUG)	+6	1st	36±5	122.95	5.12
**233**	-8.4	V (GUG)	+8	2nd	25±5	0.13	20.39
**235**	-9.5	L (CUG)	+5	4th	29±5	0	NA
**236**	-7.9	L (UUG)	+3	3rd	65±4	0.28	18.63
**238**	-8.4	V (GUG)	+4	2nd	86±5	1.54	14.86
**244**	-3.5	M (AUG)	+4	1st	360±5	272.86	3.35
**245**	-5.1	M (AUG)	+7	1st	222±11	710.51	1.23
**249**	-1.9	M (AUG)	+7	1st	391±18	98.94	5.61
**52 ΔRFP**	-1.3	M (AUG)	NA	1st	10±40*	336.19	2.89
**202 ΔRFP**	-11.0	M (GUG)	NA	2nd	28±19*	0.57	17.07
**207 ΔRFP**	-1.8	M (AUG)	NA	1st	150±21*	22.71	8.88
**214 ΔRFP**	-2.8	M (AUG)	NA	1st	264±40*	65.39	6.53
**220 ΔRFP**	-3.9	M (AUG)	NA	1st	121±29*	7011.77	-3.86
**222 ΔRFP**	-6.6	M (AUG)	NA	1st	197±28*	5.21	12.15

* background fluorescence was subtracted.

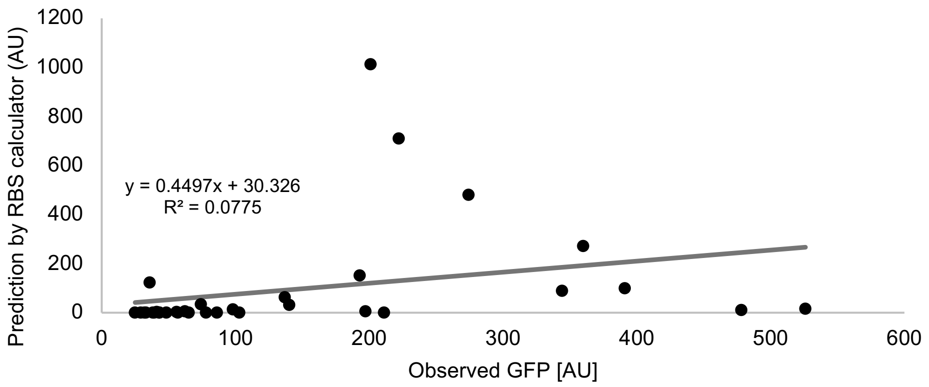

Supplementary Table 2 - Fig. 1: Correlation between observed GFP levels expressed from the synthetic operon, and those predicted upon *de novo* initiation using the RBS calculator.

Supplementary Table 3. Statistical parameters for all tests.

Test for result in figure	Test type	Alternative	Sampling position relative to stop codon[nt]	Name of sample one	N of sample two	Name of sample two*	N of sample two*	Central tendency [kCal mol^{-1} window^{-1}]	Test statistic score	P-value	Notes
**Fig. S1e**	Mann-Whitney	Two-sided	N/A	P3	266,556	P4	112,666	P3= -6.440 P4= -6.307 (means)		0.0017	
**Fig. S1e**	Mann-Whitney	One-sided: greater	N/A	P4	112,666	P5	24,104	P5= -5.996 (mean)	U= 1.4e9	$<10^{-30}$	
**Fig. S1e**	Mann-Whitney	One-sided: greater	N/A	P5	24,104	P6	147,898	P6= -5.895 (mean)	U= 1.8e9	2×10^{-6}	
**Fig. S1e**	Mann-Whitney	One-sided: greater	N/A	P6	147,898	P7	164,303	P7=-5.809 (mean)	U= 1.2e10	3×10^{-14}	
**Fig. S1e**	Mann-Whitney	One-sided: greater	N/A	P7	164,303	P8	259,634	P8= -5.712 (mean)	U= 2.2e10	$<10^{-30}$	
**Fig. 2b**	Wilcoxon	Two-sided	+5	Sample	82,360 (4,118 genes)	N/A	N/A	-2.37 (mean)	W=1.1e10	$<10^{-30}$	Was compared under the RTS model (see methods)
**Fig. 2d**	Mann-Whitney	Two-sided	+4	High protein abundance	24,440	Low and medium protein abundance	57,000	-2.0 (median)	U=5.8e8	$<10^{-30}$	
**Fig. 3b**	Wilcoxon	Two-sided	0	<25	1,537	N/A	N/A	0.4 (mean)	W= 7.5e5	5×10^{-19}	
**Fig. 3b**	Wilcoxon	Two-sided	0	≥25	2,581	N/A	N/A	-4.0 (mean)	W= 5.9e5	$<10^{-30}$	
**Fig. 3c**	Mann-Whitney	Two-sided	0	≥25	51,620	<25	30,920	-2.0 (median)	U= 4.8e8	$<10^{-30}$	
**Fig. 4a**	Mann-Whitney	Two-sided	+3	Last genes	45,500	Not last genes	33,740	-2.3 (median)	U= 5.0e8	$<10^{-30}$	

*Two-sample testing

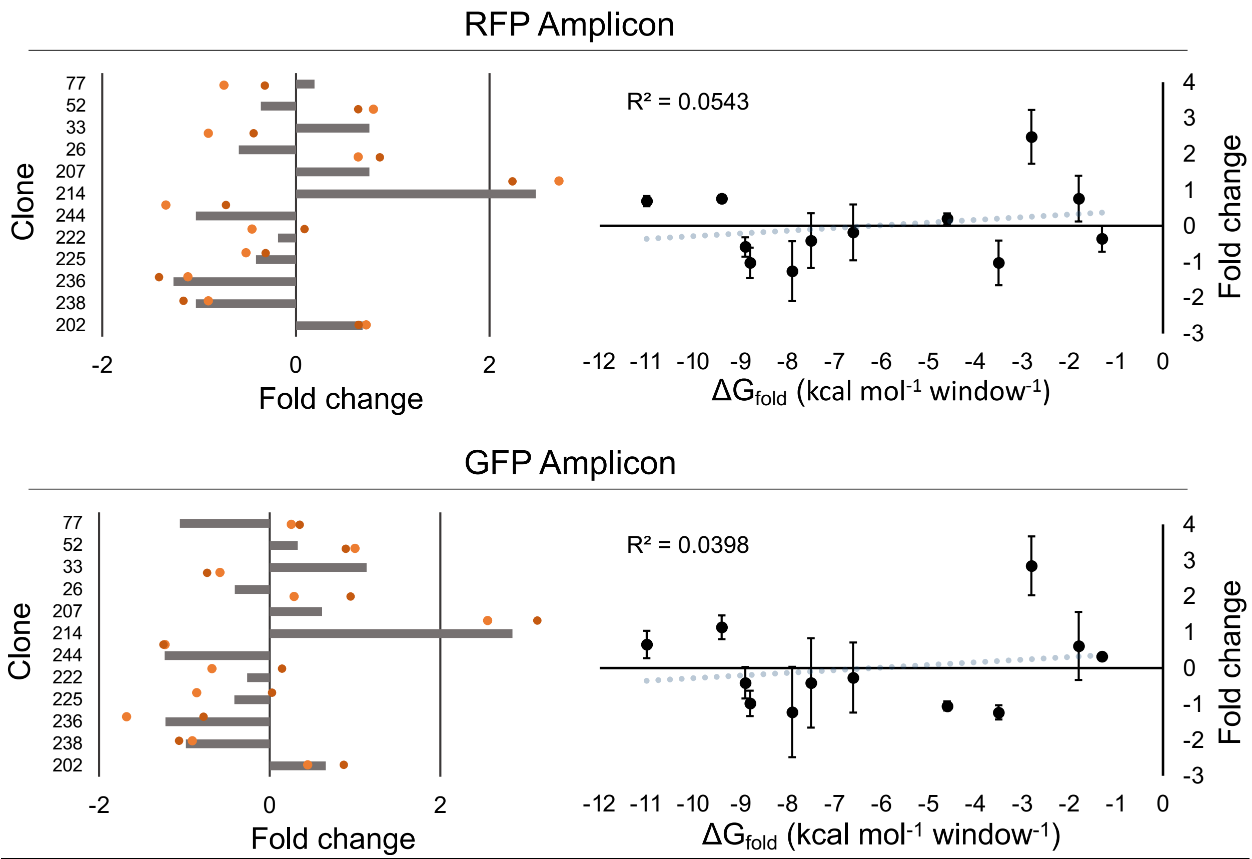

Supplementary Figure 2 – Quantitative PCR of synthetic operon mRNA levels. mRNA abundance fold change (left) measured by two experimental repeats of qPCR, each with two or three replications of twelve select clones, including the eight clones from the subgroup described in Fig. 1f. Fold change is relative to the average mRNA abundance of all clones. No significant correlation was noted between ΔG_{fold} of the variable region in several pRNXG clones and mRNA abundance in *E. coli* MG1655 (scatter plots; right), error bars represent a standard deviation of the mean. This was confirmed with amplicons of regions up-stream (RFP amplicon) and down-stream (GFP amplicon) of the variable sequence region. All amplicons were normalized to 16S rRNA amplicon abundance, and the primer efficiencies were >99%. The no-template controls (NTC) quantitation cycles (CQ) were at least 15 cycles larger than samples.

We realize that changes that are below two-fold cannot be accurately determined using qPCR while still having a significant effect on protein expression levels. However, one should consider that the changes in protein expression levels were observed only for GFP and not for RFP genes. This means that if the RTS effect stems from differences in mRNA abundance, it is likely only through differential degradation of GFP message in the mRNA molecule. We do not see such differential degradation, nor are we aware of a mechanism that will support such degradation that will correlate with our results.

In addition, GFP expression level's dynamic range is ~16-fold between the clones, and the expression levels of those clones with a strong RTSs (such as clone 202), is negligible (the level of negative controls which express only the RFP gene and are undetectable in Western blots). If this basal GFP expression effect is a result of differential degradation of mRNA, we think that it should be a result of larger than a two-fold

change between a non-producer and a high producer (such as clone 207). Also, we think that it should have been even more probable to capture a weak correlation between ΔG_{fold} and mRNA abundance if it was the source of the effect. However, there was no such correlation (r^2 values of 0.05 and 0.04 for RFP and GFP amplicons, respectively).

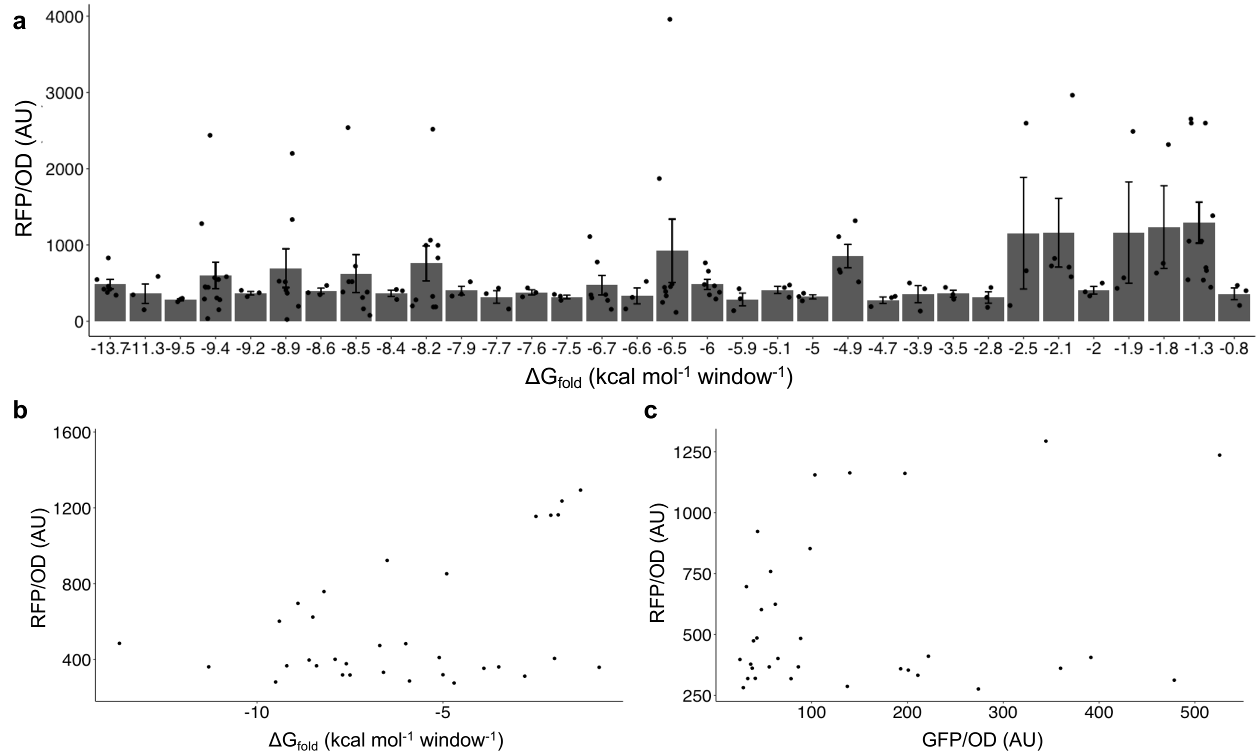

Supplementary Figure 3. RFP expression from different synthetic operon clones. a) **Mean** expression levels of RFP normalized to OD_{600} measured by RFP fluorescence; error bars represent standard error of experimental repeats, the number of experimental repeats for each clone is represented by the number of points scattered, but for all clones, at least three measurements were taken (n≥3). **b**) Correlation between RFP fluorescence levels and ΔG_{fold}. No significant correlation was observed (Spearman correlation=-0.19, S=7,118, n=33, p-value=0.29). **c**) Dependence between GFP and RFP expression levels of the synthetic operon. No significant correlation was observed (Spearman correlation=0.08, S=5,528 p-value=0.67).

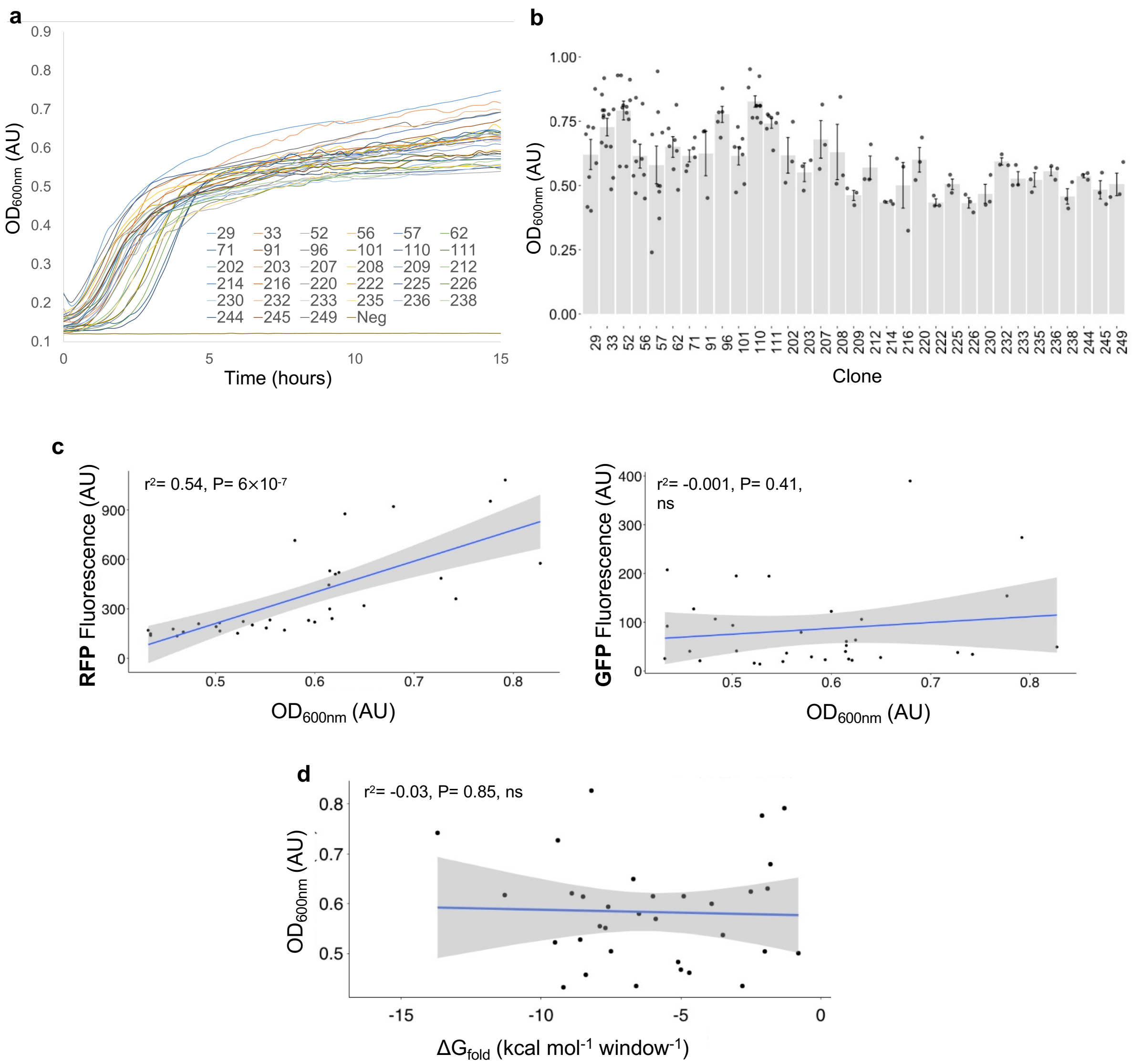

Supplementary Figure 4. Bacterial growth rates of isolated library clones. a) Representative bacterial growth curves, presenting the average OD_{600} over time of n=3 technical replicates, for all clones used in this study. **b)** The average maximal OD_{600} achieved by each clone, error bars represent the standard error of each clone. **c)** The left panel presents the linear Fischer correlation between RFP levels, and bacterial growth was found to be significant regardless of the clone-specific genotype (n=33, F=39.11, adjusted r^2=0.54, P-value= 5.978e^{-07}). The right panel presents the linear Fischer correlation between GFP levels, and bacterial growth was found to be non-significant. This can be interpreted as the effect of each clone-specific genotype on GFP expression is more substantial than the contribution of bacterial density (n=33, F=0.7106, adjusted r^2=-0.001, P-value= 0.41). **d)** The linear Fischer correlation between bacterial growth and ΔG_{fold} of the variable sequence of each clone was found to be non-significant (n=33, F=0.04, adjusted r^2=-0.03, P-value= 0.8466).

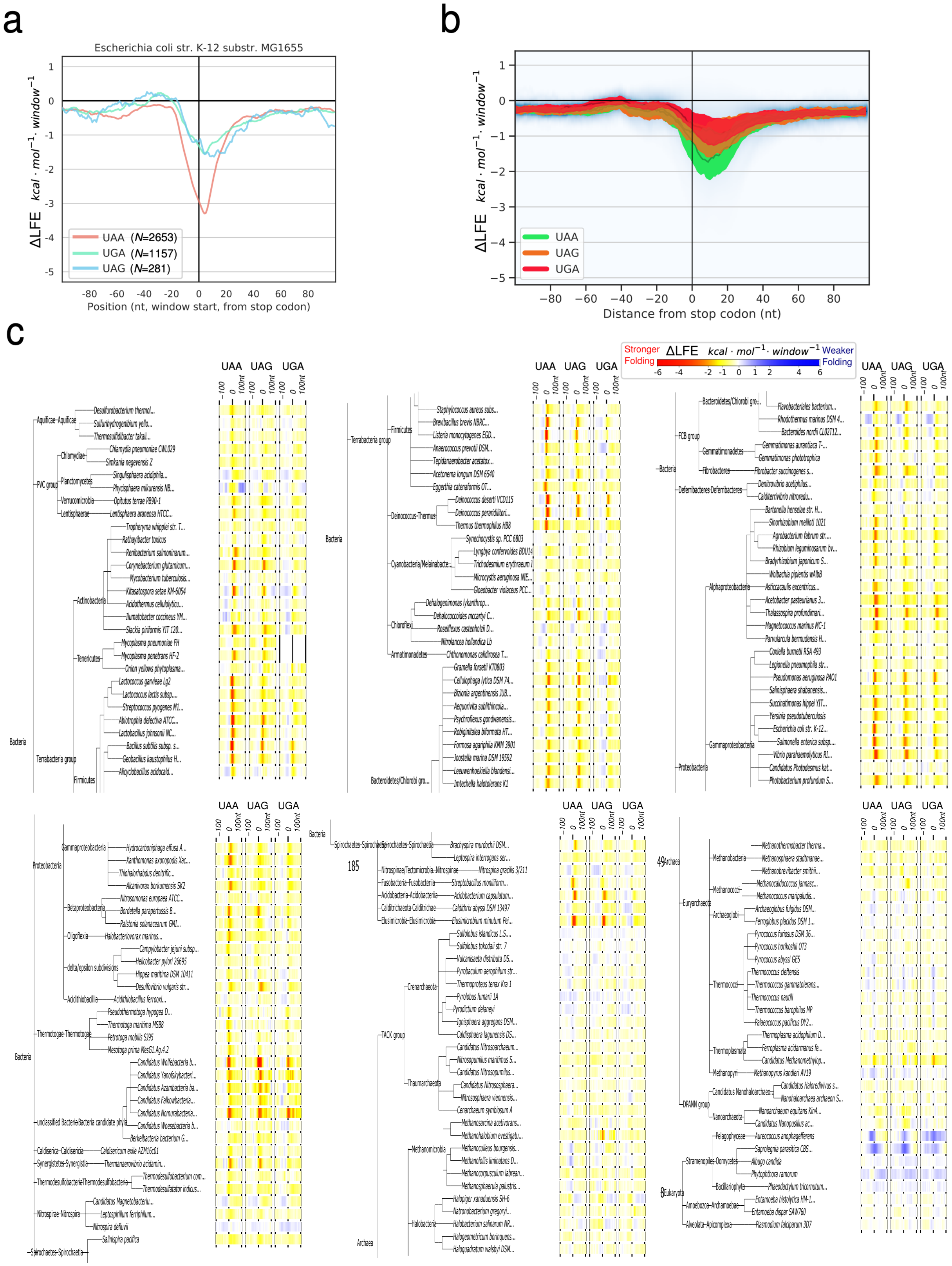
a
Escherichia coli str. K-12 substr. MG1655
ΔLFE kcal · mol⁻¹ · window⁻¹
Position (nt, window start, from stop codon)
UAA (N=2653)
UGA (N=1157)
UAG (N=281)
b
ΔLFE kcal · mol⁻¹ · window⁻¹
Distance from stop codon (nt)
UAA
UAG
UGA
c
Stronger Folding
ΔLFE kcal · mol⁻¹ · window⁻¹
Weaker Folding
UAA UAG UGA
Bacteria
Archaea
Eukaryota

Supplementary Figure 5. ΔLFE of the three stop codons in E. coli and 128 other bacterial strains. a) All three stop codons were tested independently for RTS in *E. coli* presence and strength. While the RTS was present after all stop codons, differences were observed. **b**) Where examined, RTS presence and stop codon-related differences in 128 bacterial species were, on average, consistent with what was observed in *E. coli.* In addition, ΔLFE heatmaps depicting the 100 nucleotide-long regions around stop codons across the tree of life for each of the three stop codons (warm colors: stronger folding than expected; cool colors: weaker folding than expected) was drawn. **c**) 128 bacterial, 49 archaeal, and 8 eukaryotic species were examined. The two latter domains presented mixed results.

a

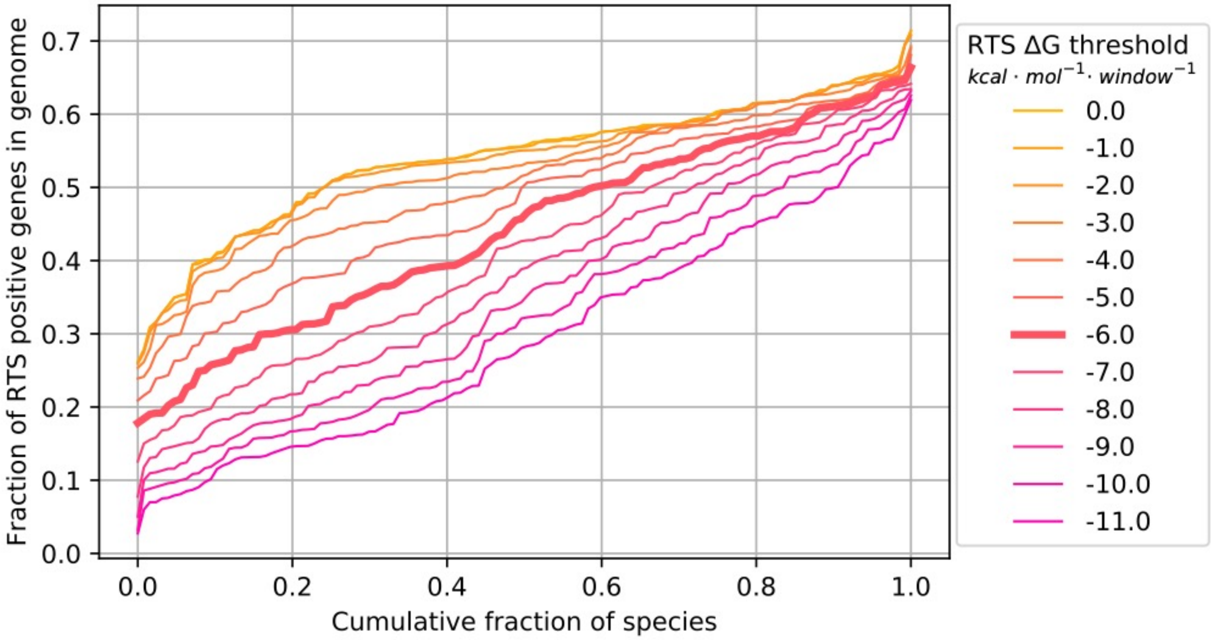

Supplementary Figure 6a. RTS presence across all kingdoms of life (all stop codons aggregated). Parameter sensitivity and effect of different **Δ**G thresholds on the number of RTS containing genes, under the RTS model (see methods), for all bacteria (*N*=128). The selected threshold value of -6.0 kcal mol^{-1} $window^{-1}$, for the heat maps presented in Fig. 2f, and Fig. S6b is highlighted.

b

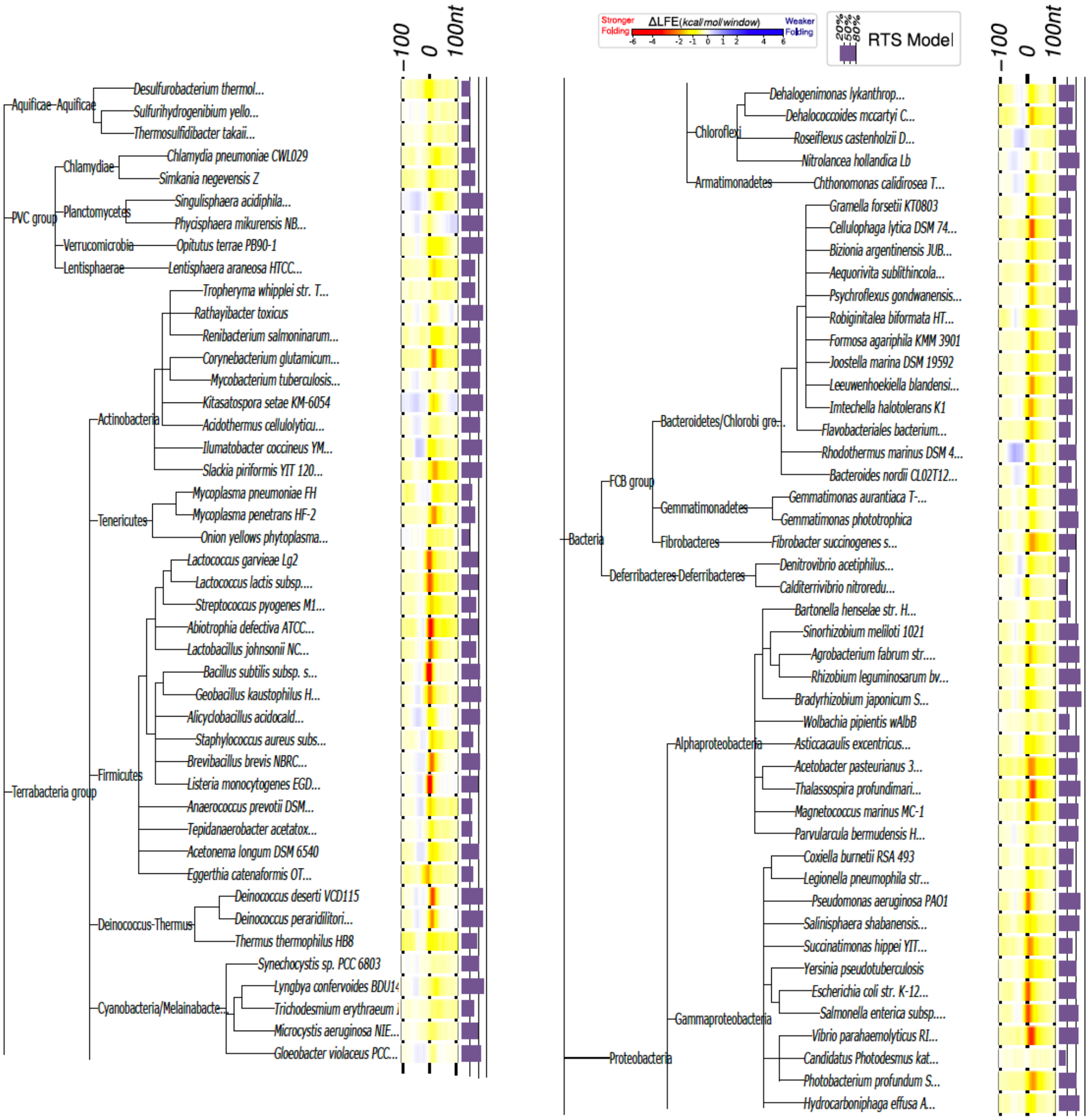

-100
0
100nt
Stronger Folding
ΔLFE(kcal/mol/window)
Weaker Folding
-6 -4 -2 -1 0 1 2 4 6
20%
50%
80%
RTS Model
Aquificae-Aquificae
Desulfurobacterium thermol...
Sulfurihydrogenibium yello...
Thermosulfidibacter takaii...
PVC group
Chlamydiae
Chlamydia pneumoniae CWL029
Simkania negevensis Z
Planctomycetes
Singulisphaera acidiphila...
Phycisphaera mikurensis NB...
Verrucomicrobia
Opitutus terrae PB90-1
Lentisphaerae
Lentisphaera araneosa HTCC...
Terrabacteria group
Actinobacteria
Tropheryma whipplei str. T...
Rathayibacter toxicus
Renibacterium salmoninarum...
Corynebacterium glutamicum...
Mycobacterium tuberculosis...
Kitasatospora setae KM-6054
Acidothermus cellulolyticu...
Ilumatobacter coccineus YM...
Slackia piriformis YIT 120...
Tenericutes
Mycoplasma pneumoniae FH
Mycoplasma penetrans HF-2
Onion yellows phytoplasma...
Firmicutes
Lactococcus garvieae Lg2
Lactococcus lactis subsp....
Streptococcus pyogenes M1...
Abiotrophia defectiva ATCC...
Lactobacillus johnsonii NC...
Bacillus subtilis subsp. s...
Geobacillus kaustophilus H...
Alicyclobacillus acidocald...
Staphylococcus aureus subs...
Brevibacillus brevis NBRC...
Listeria monocytogenes EGD...
Anaerococcus prevotii DSM...
Tepidanaerobacter acetatox...
Acetonema longum DSM 6540
Eggerthia catenaformis OT...
Deinococcus-Thermus
Deinococcus deserti VCD115
Deinococcus peraridilitori...
Thermus thermophilus HB8
Cyanobacteria/Melainabacte...
Synechocystis sp. PCC 6803
Lyngbya confervoides BDU1
Trichodesmium erythraeum
Microcystis aeruginosa NIE...
Gloeobacter violaceus PCC...
Bacteria
Chloroflexi
Dehalogenimonas lykanthrop...
Dehalococcoides mccartyi C...
Roseiflexus castenholzii D...
Nitrolancea hollandica Lb
Armatimonadetes
Chthonomonas calidirosea T...
FCB group
Bacteroidetes/Chlorobi gro...
Gramella forsetii KT0803
Cellulophaga lytica DSM 74...
Bizionia argentinensis JUB...
Aequorivita sublithincola...
Psychroflexus gondwanensis...
Robiginitalea biformata HT...
Formosa agariphila KMM 3901
Joostella marina DSM 19592
Leeuwenhoekiella blandensi...
Imtechella halotolerans K1
Flavobacteriales bacterium...
Rhodothermus marinus DSM 4...
Bacteroides nordii CL02T12...
Gemmatimonadetes
Gemmatimonas aurantiaca T-...
Gemmatimonas phototrophica
Fibrobacteres
Fibrobacter succinogenes s...
Deferribacteres-Deferribacteres
Denitrovibrio acetiphilus...
Calditerrivibrio nitroredu...
Proteobacteria
Alphaproteobacteria
Bartonella henselae str. H...
Sinorhizobium meliloti 1021
Agrobacterium fabrum str....
Rhizobium leguminosarum bv...
Bradyrhizobium japonicum S...
Wolbachia pipientis wAlbB
Asticcacaulis excentricus...
Acetobacter pasteurianus 3...
Thalassospira profundimari...
Magnetococcus marinus MC-1
Parvularcula bermudensis H...
Gammaproteobacteria
Coxiella burnetii RSA 493
Legionella pneumophila str...
Pseudomonas aeruginosa PAO1
Salinisphaera shabanensis...
Succinatimonas hippei YIT...
Yersinia pseudotuberculosis
Escherichia coli str. K-12...
Salmonella enterica subsp....
Vibrio parahaemolyticus RI...
Candidatus Photodesmus kat...
Photobacterium profundum S...
Hydrocarboniphaga effusa A...

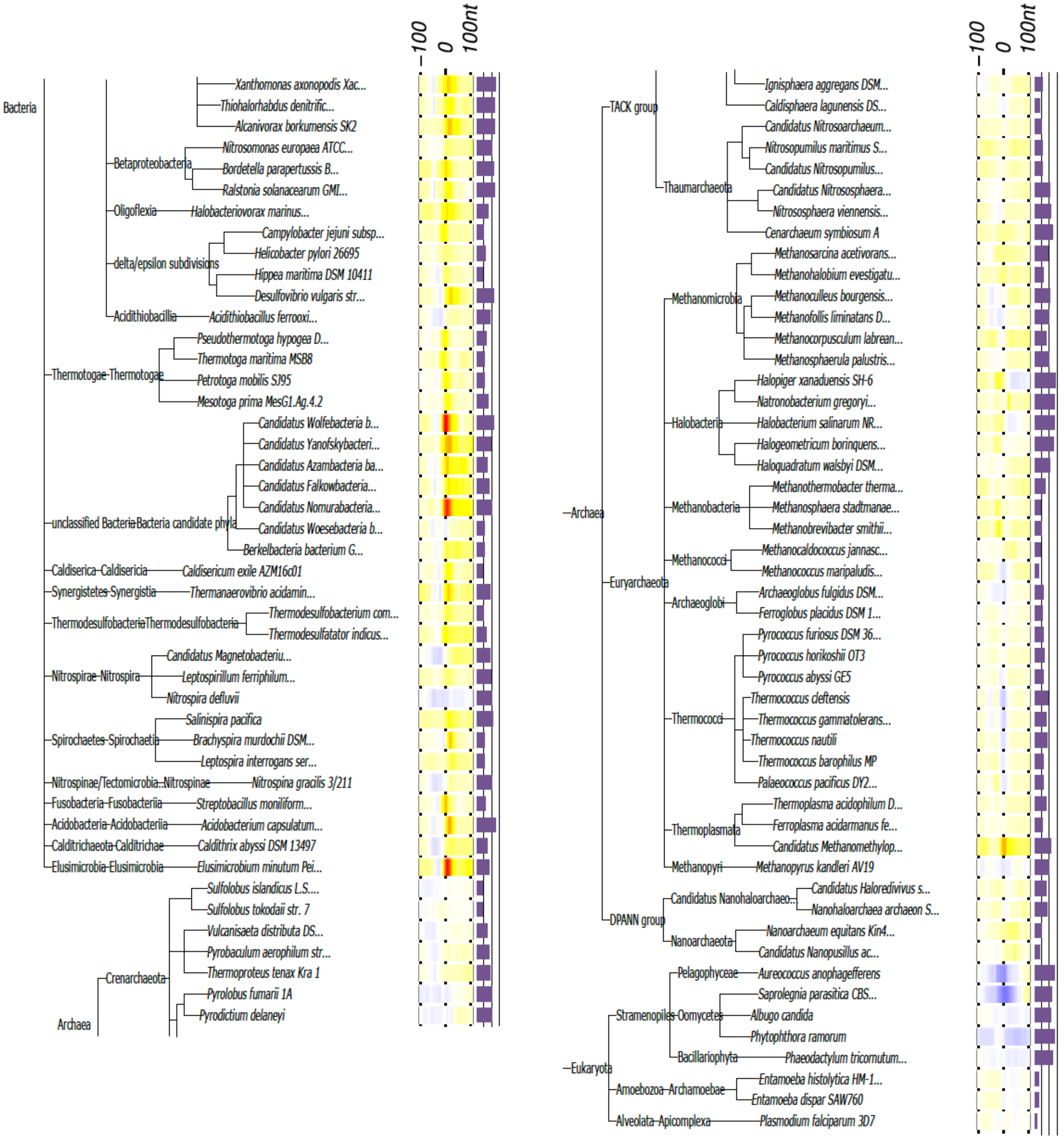

Supplementary Figure 6b. ΔLFE landscape of in 128 bacteria, 59 archaea, and 8 eukaryotes. The ΔLFE landscape was depicted as a heatmap of 100 nucleotide-long regions around stop codons in species belonging to domains comprising the three branches of the tree of life (warm colors: stronger folding than expected; cool colors: weaker folding than expected). Using the RTS model (see Methods), we assessed the presence or absence of the RTS. The results revealed that 122/128 (95.3%) of bacteria, 12/49 (24.5%) of archaea and 2/8 (25.0%) of eukaryotes present an apparent RTS, although the sample sizes of the two latter groups are too small and the RTS signal is too weak and unreliable to draw any conclusions at this time. Raw data of native LFE and ΔLFE profiles for all species are included in supplementary data tables 5a-c.

a

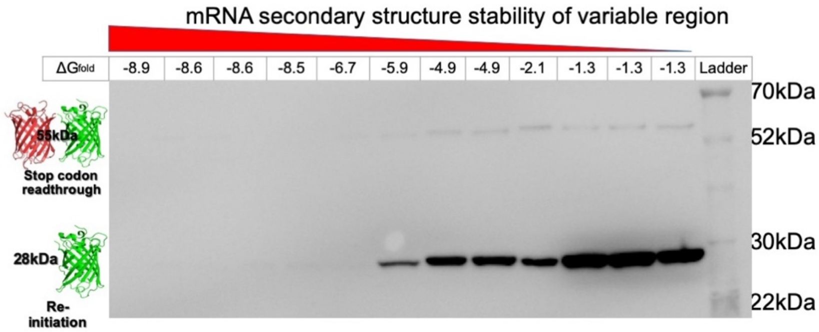

b

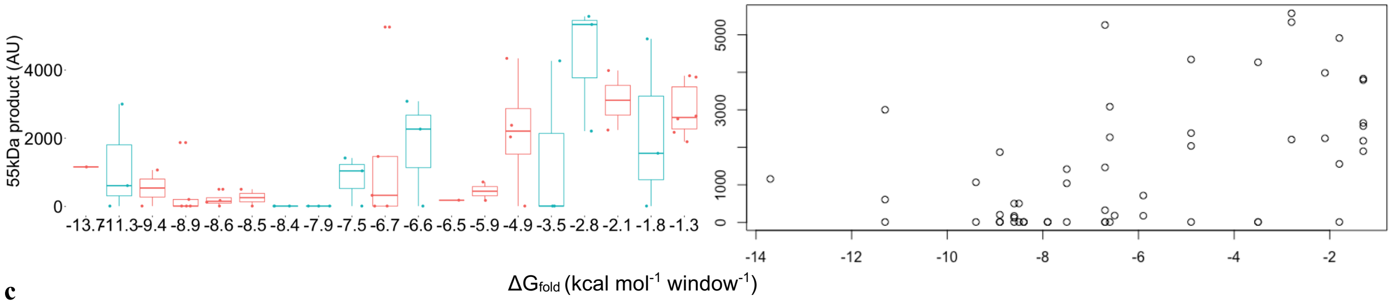

c

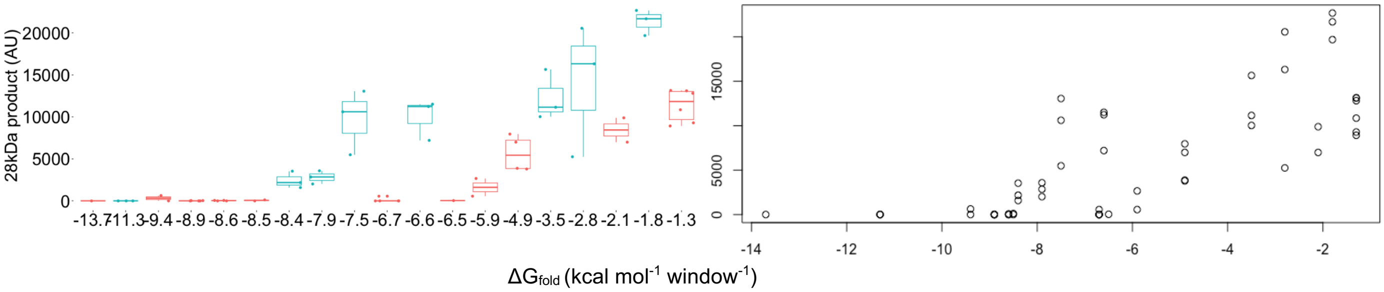

Supplementary Figure 7. Densitometry analyses of Western blots. a) Anti-His tag Western blot of random clones. For the randomly selected clones (red) and for the clones with an AUG start codon beginning at positions +3 or +4 (cyan), both **b)** the 55 kDa RFP-GFP product resulting from stop codon read-through, and **c)** the 28 kDa GFP product resulting from *de novo* initiation or re-initiation were measured using densitometry of the pRXNG clones in *E. coli* MG1655. The results were aggregated experimental repeats of each close as a box-plot for each clone (left) and as scatterplots for correlation analyses. In the scatterplots, each data point represents one experimental anti-His tag Western blot repeat of a clone with the indicated calculated ΔG_{fold}. The 28 kDa GFP product accounts for 91% of the correlation between ΔG_{fold} and the total amount of GFP expressed by the different clones (omega squared test, ω^2=0.91). Moreover, correlation with ΔG_{fold} was maintained for GFP (Spearman correlation, ρ=0.80, n=58, S=6479, p-value=4.537e-14) and also, albeit to a lesser degree, with the 55 kDa read-through product (Spearman correlation ρ=0.50, n=58, S=16326, p-value=7.011e-5).

Clone 52:

ESI Mass Spectrum:

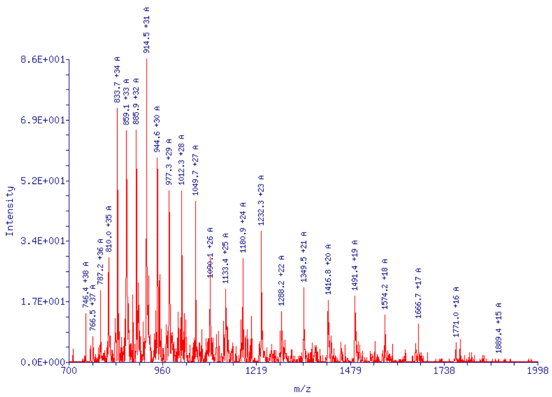

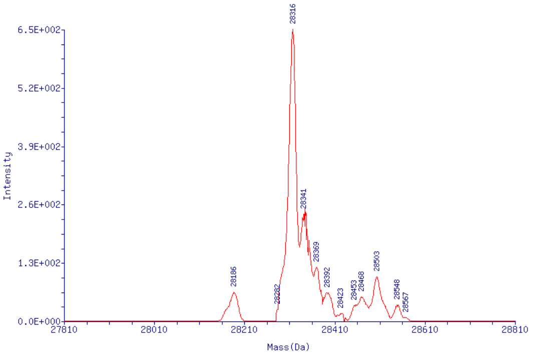

Clone 71:

ESI Mass Spectrum:

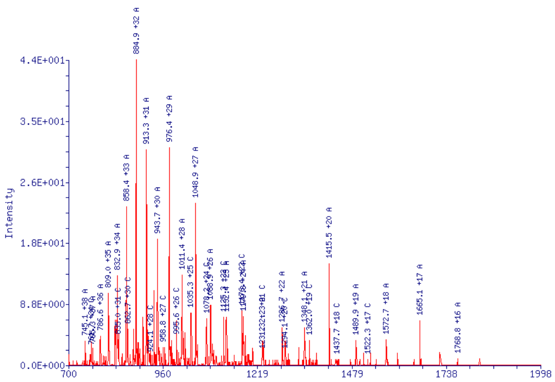

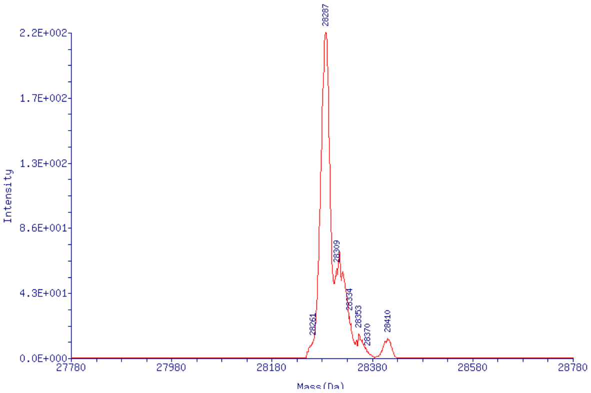

Clone 91:

ESI Mass Spectrum:

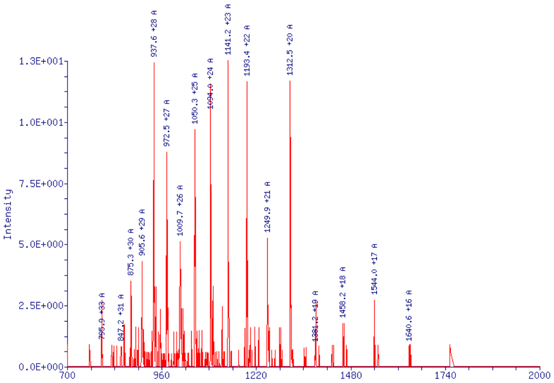

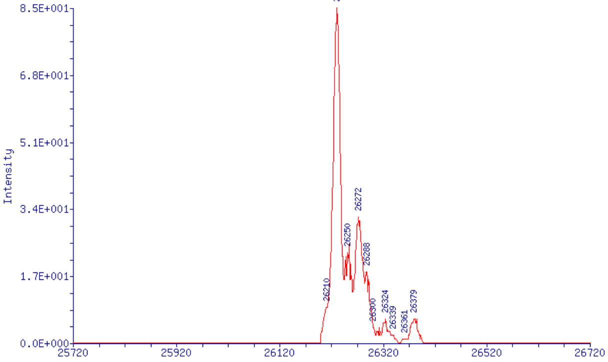

Clone 96:

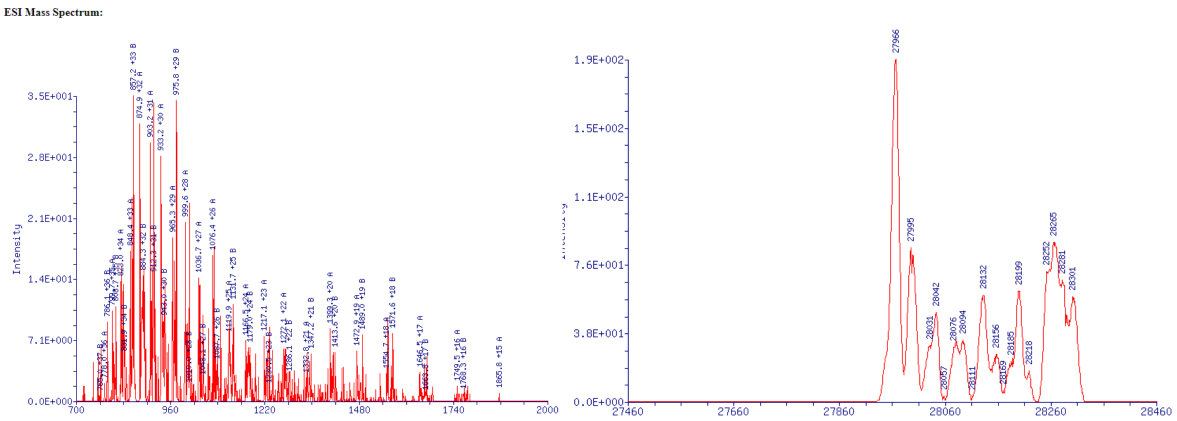

Clone 110:

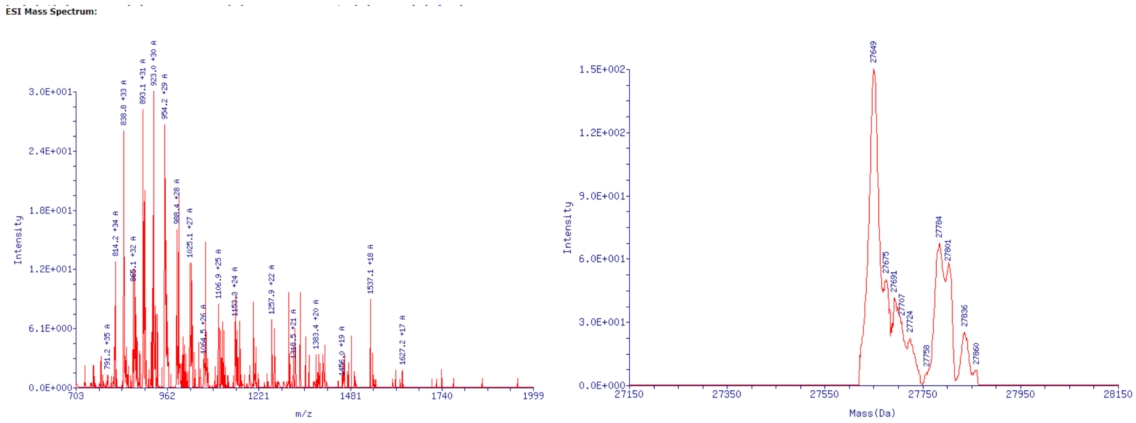

Full read-through product (entire dual reporter construct):

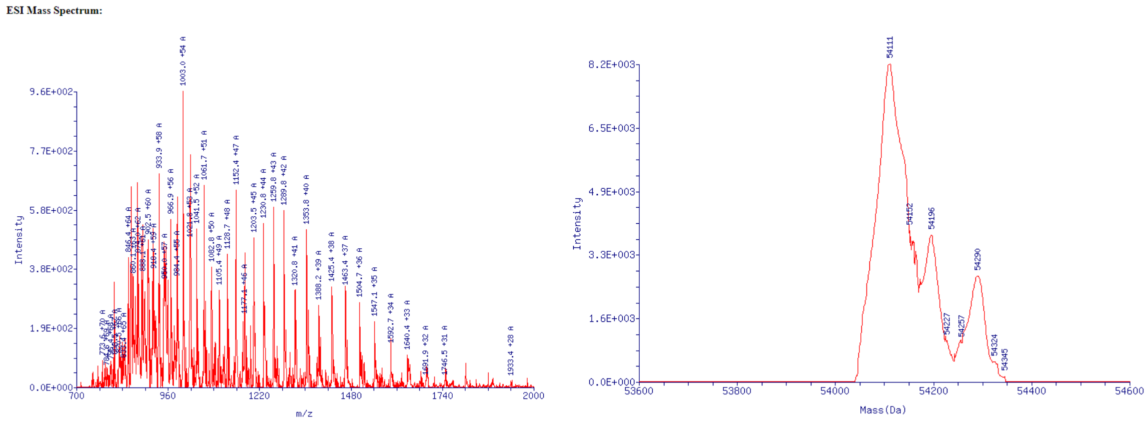

Supplementary Figure 8. Mass spectra of different clones. Five clones expressing sufficient levels of the ~28 kDa GFP product and a representative read-through product (with the UAG stop codon mutated to encode tyrosine) were purified using nickel affinity columns and subjected to mass spectrometry to identify the start codon. These involved comparisons of calculated masses generated by the clone-specific sequence and the measured mass of the protein. Left panels depict the raw MS results, while the right panels depict de-convoluted data obtained using Promass software. In the manuscript, we report the primary product of each clone. However, we cannot exclude or accurately assess the possibility of multiple possible initiation sites with different efficiencies.

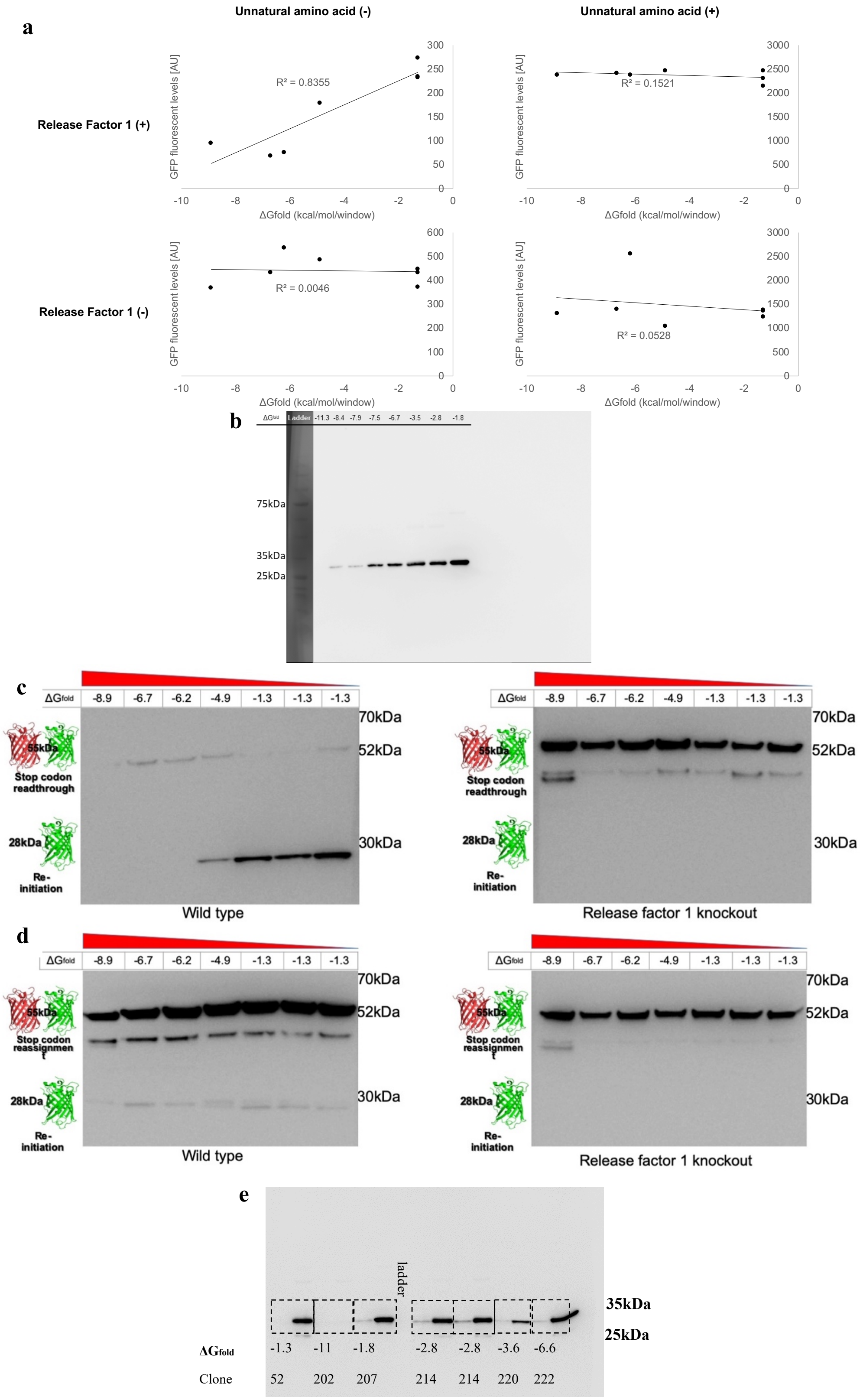
a
Unnatural amino acid (-)
Unnatural amino acid (+)
Release Factor 1 (+)
Release Factor 1 (-)
GFP fluorescent levels [AU]
ΔGfold (kcal/mol/window)
R² = 0.8355
R² = 0.1521
R² = 0.0046
R² = 0.0528
b
Ladder
75kDa
35kDa
25kDa
c
ΔGfold
-8.9 -6.7 -6.2 -4.9 -1.3 -1.3 -1.3
70kDa
52kDa
30kDa
55kDa
Stop codon readthrough
28kDa
Re-initiation
Wild type
Release factor 1 knockout
d
ΔGfold
-8.9 -6.7 -6.2 -4.9 -1.3 -1.3 -1.3
70kDa
52kDa
30kDa
55kDa
Stop codon reassignment
28kDa
Re-initiation
Wild type
Release factor 1 knockout
e
ladder
35kDa
25kDa
ΔGfold
-1.3 -11 -1.8 -2.8 -2.8 -3.6 -6.6
Clone
52 202 207 214 214 220 222

Supplementary Figure 9. Correlation between ΔGfold and GFP levels without and with Release Factor 1 (RF1). **a**) Comparison of GFP expression, measured by fluorescence, between *E. coli* C321.ΔprfA EXP and MG1655, both transformed with the pEVOL pylRS genetic code expansion system and five pRXNG library clones with different ΔG_{fold}. Each data point represents the average of n=3 experimental replicates. **b**) Uncropped anti-His-tag Western blots presented in Fig. 3e of eight pRXNG clones with AUG start codon in the 3rd of 4th codon downstream from the RFP stop codon. This experiment was repeated independently with similar results 4 times **c**) Uncropped anti-His-tag Western blots presented in Fig. 3g of five pRXNG library clones with different ΔG_{fold}. This experiment was repeated independently with similar results 4 times **d**) Uncropped gels presented in Fig. 3h. The bands below the RFP-GFP product (with a size of ~50kDa) are the his-tagged pyrrolysyl synthetase (*pylRS)* gene from the co-transformed pEVOL plasmid which is used for genetic code expansion is transformed. This experiment was repeated independently with similar results four times **e**) The uncropped blot of Fig. 3i. This experiment was repeated independently once.

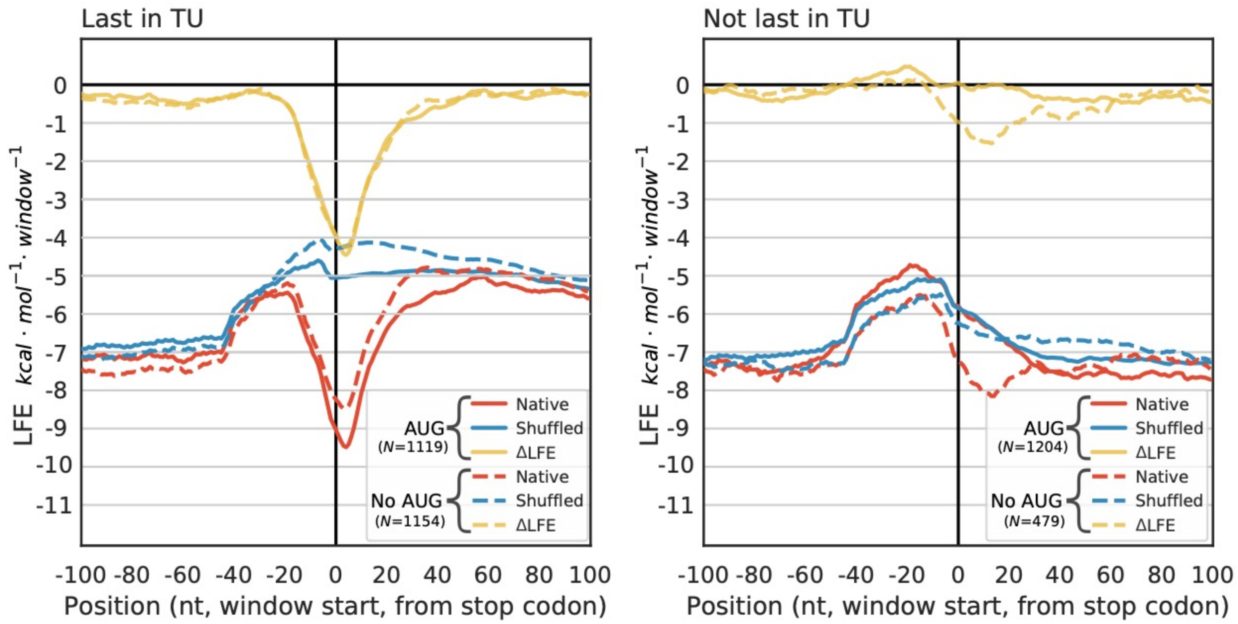

Supplementary Figure 10. Analyses of operonic position effect on RTS presence with/without a down-stream AUG start codon. Terminal operonic genes either with or without an AUG start codon in-frame of the down-stream CDS in the 50 nt downstream of a stop codon. Right panel: Mid-operonic genes either with or without an AUG start codon in-frame of the down-stream CDS in the 50 nt downstream of a stop codon. We examined differences between two groups of genes, namely those assuming the last position in an operon (i.e., terminal genes) **(left)** versus all other operon genes (i.e., non-terminal genes) **(right)**. Each group was further divided according to the presence of an in-frame AUG start codon within 50 nt downstream of the stop codon or the absence of a start codon. Such divisions revealed that in terminal genes, where translation insulation is expected in all cases, significant selection for an RTS was observed, regardless of the presence or absence of a down-stream start codon. Conversely, in mid-operon genes, selection for RTSs in the group with the start codon, where re-initiation is expected, is not higher than random. In the second group, where re-initiation is not desired as no in-frame AUG start codon exists, significant selection for RTSs was observed.

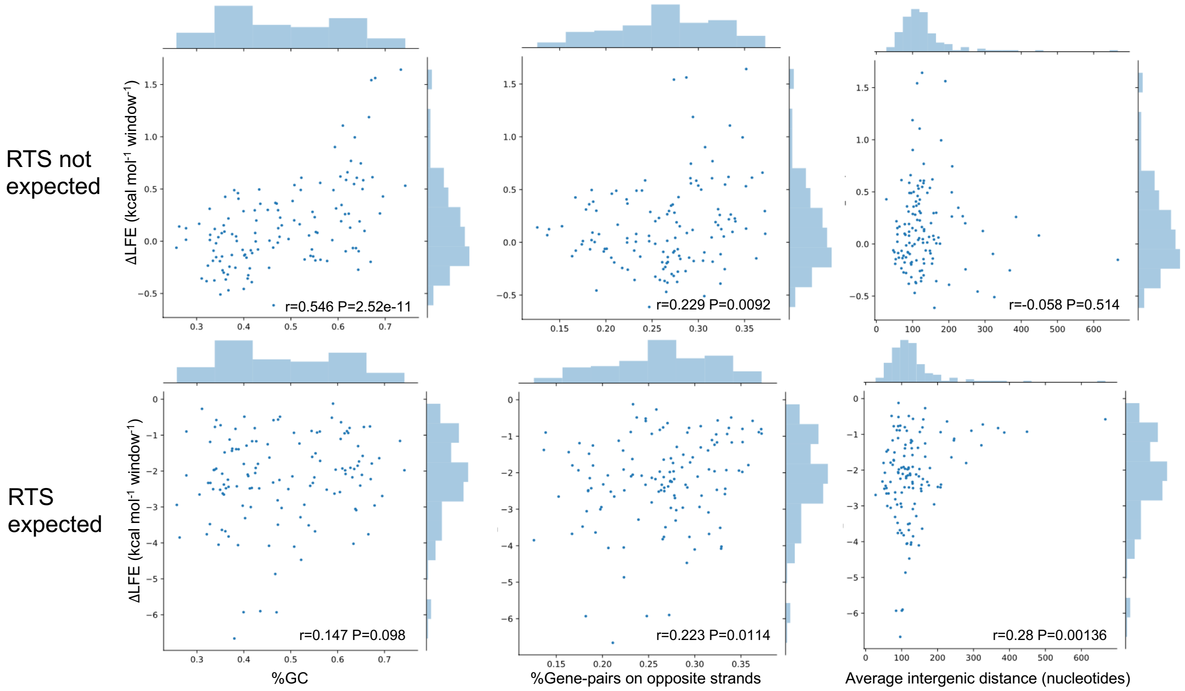

Supplementary Figure 11. Genomic traits explain some of the variability in selection strength for RTS between species. Correlation between three genomic traits and ΔLFE (i.e., the strength of selection for the RTS) across 128 bacterial strains. Each dot represents one bacterial strain. r values and statistical significance are calculated using the Pearson correlation (n=128).

For each of the 128 bacterial species examined herein, all genes were separated into two groups following these conditions: Group 1) Genes with downstream intergenic distances of less than 25 nucleotides to the next CDS and are on the same strand. In this group, RTS is less expected, and enrichment of mid-operonic genes is expected. Group 2) Genes with a downstream intergenic distance of more than 25 nucleotides to the next CDS or are on opposite strands of the DNA. Three genomic traits where explored: a) %GC content, the proportion of GC in the genome (i.e., %GC). b) The proportion of genes in the genome, which are followed by a downstream gene on an opposite strand; this measure is used as a proxy to the length and number of operons in the species genome. And c) The average intergenic distance between all genes in a species genome. This measure is used as a proxy to the compression of the host genome, which is suspected of having implications regarding the usage, number, and size of operons.

The mean ΔLFE around the stop codons of all genes in each species was calculated, and the minimum ΔLFE found in the region between -10nt and 20nt relative to the first nucleotide of the 3'-UTR, was used as the ΔLFE value for each species.

We controlled for the fact that a stable mRNA structure down-stream of a stop codon could be functionally related to transcription termination since rho-independent transcription terminators can form stable mRNA hairpins. Therefore, to distinguish the role of the RTS in regulating translation re-initiation from transcription termination, all 871 known or suspected genes that terminate with a rho-independent terminator sequence[3] were removed from the analysis (Fig. S10, left). The RTS signal remained (Wilcoxon test, p-value$<10^{-16}$). The reduction in the effect is probably due to the fact that Rho-independent terminators affected the analysis by biasing the sequences ~40-60 nt downstream of the stop codon to more stable structures, thus interfering with our analysis around the stop codon, as the window size used was 40 nt. To further demonstrate the absence of a link between the RTS and transcription termination, two subsets of the terminal and monocistronic genes were analyzed according to their experimentally measured 3' UTR lengths[4] (Fig. S10, right), with one group presenting short 3' UTRs (<50 nt) and the other possessing long 3' UTRs (>50 nt). If the RTS signal is linked to transcription termination, one would expect to see the RTS signal closer to the stop codons in the former and further away from the stop codon in the latter. However, no change in the position or magnitude of the RTS was observed. These analyses, taken together, demonstrate that the RTS is not linked to transcription termination.

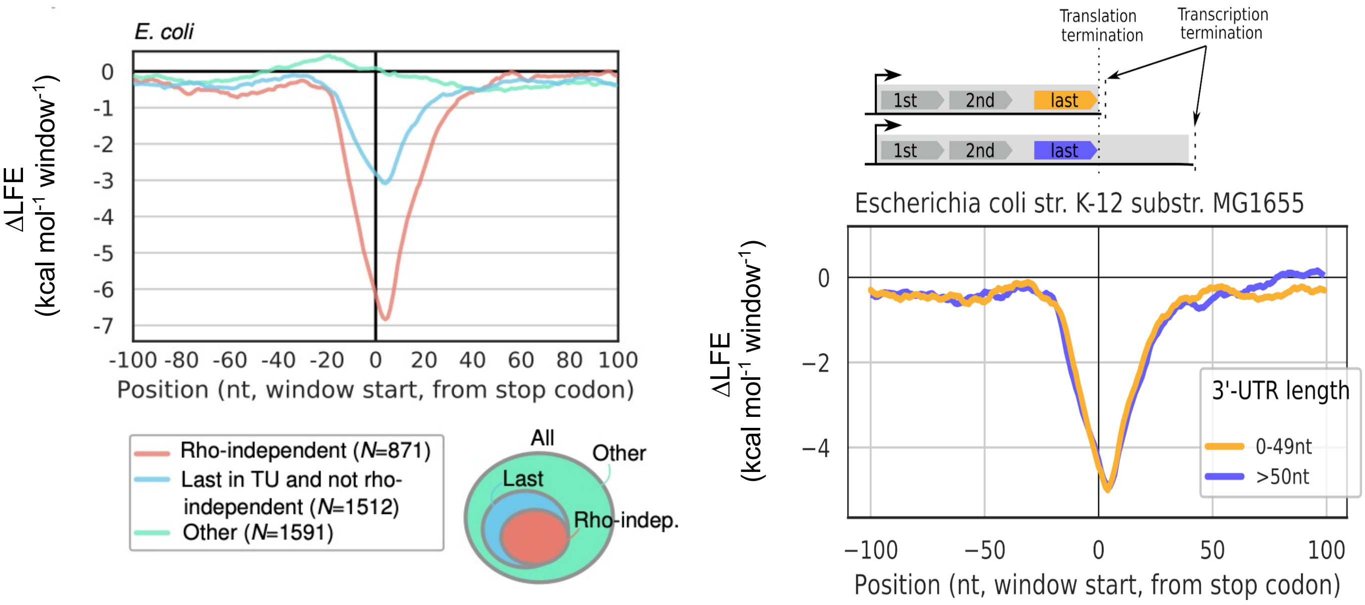

Supplementary Figure 12. Controlling for an RTS link to transcription termination. left panel) Analyses of *E. coli* genes grouped by transcription termination mechanism shows that folding bias cannot be explained by the presence of rho-independent terminators. Red, genes with rho-independent terminators. Blue, genes that are last in their transcription units (TU) but do not have rho-independent terminators. Green, all other genes. Lines represent ΔLFE, computed as described in the Methods section. Annotation of rho-independent genes based on WebGesTer-DB. Annotation of TU positions based on the ODB4 database. **Right panel)** The RTS signal shows no change between groups of genes with short (<50 nt) or long (>50 nt) 3' UTRs.

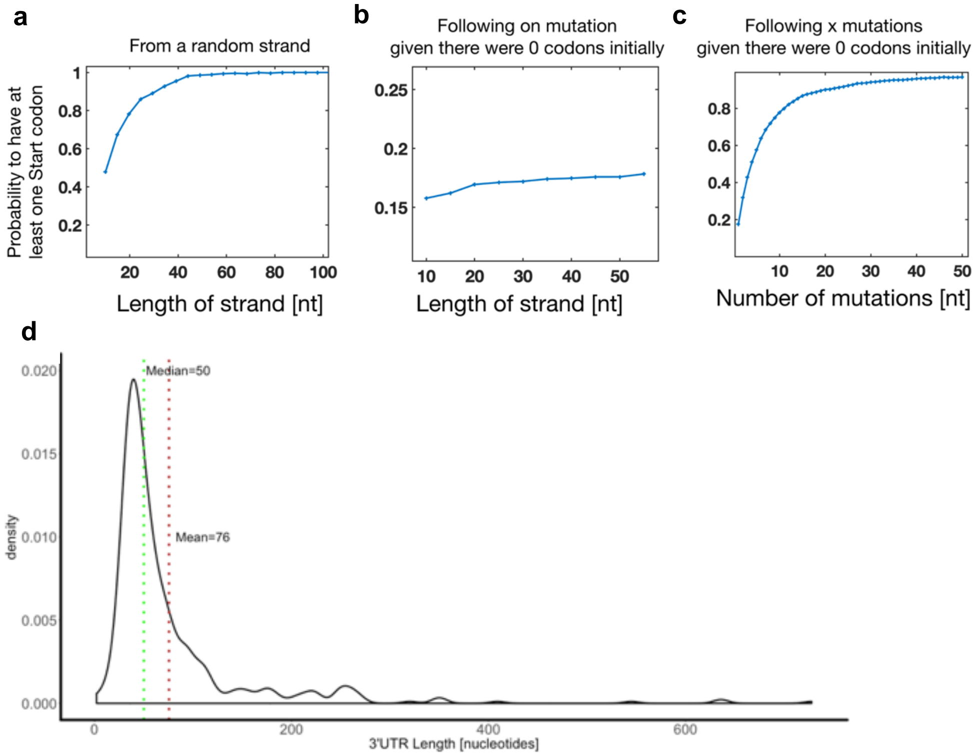

Supplementary Figure 13. Probability of having a start codons downstream of a stop codon without selection. **a**) The probability of having at least one efficient start codon (ATG, GTG, TTG, CTG, ATA, ATT) by chance as a function of DNA length. **b**) The probability that a sequence with no efficient start codon will generate an efficient start codon after a one nucleotide mutation as a function of strand length (Juke and Cantor, one parameter mutation model). **c**) The probability of having at least one efficient start codon through consecutive mutations on a fixed, 50 base pair-long DNA stretch. **d**) Density plot of mapped *E. coli* 3' UTR lengths in the RegulonDB database[4] (470 transcriptions units).

When considering the evolution of translation re-initiation, two solutions to avoid un-intended re-initiations when this is deleterious (for example, after the last gene of a poly-cistronic mRNA) are possible. The first involves depleting all efficient start codons. However, this is not optimal for three reasons: i) Even inefficient start codons could lead to basal expression by re-initiation; ii) ribosomes would wastefully spend time scanning for start codons which are depleted, resulting in a fitness cost; and iii) the probability of efficient start codons (one of the six most efficient[1]) on a random 3'UTR sequence is >0.9 (Fig. S11a) if considering the median *E. coli* 3' UTR length of 50 nucleotides (Fig. S11d). Moreover, the selection on the 3' UTR would have to be extremely high to counter the ~17% chance of an efficient start codon appearing after each single nucleotide mutation (Fig. S11b). This constraint is further compounded by consecutive mutations (Fig. S11c). To assess the length of *E. coli* 3'UTRs, we utilized RNA-seq data[4]. The data revealed that in *E. coli,* the average 3' UTR length is 76 nucleotides, with the median length being 50 nucleotides, a sufficient length to harbor significant mRNA secondary structure, and require stringent selection to avoid start codon-generated mutations.

Putative RTS sequences

	Logo	E-value	Sites
1.	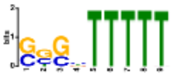	2.2e-050	359
2.	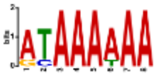	3.6e-007	148
3.	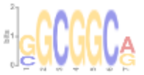	1.5e-001	100
4.	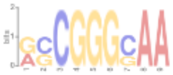	2.7e+003	22
5.	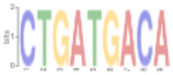	1.1e+004	13
6.	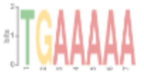	2.5e+004	92
7.	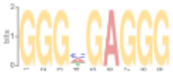	1.2e+005	20
8.	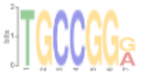	1.3e+005	83
9.	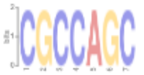	1.1e+005	47
10.	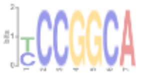	1.4e+005	82

Putative non-RTS sequences

	Logo	E-value	Sites
1.	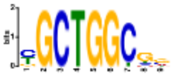	2.8e-015	95
2.	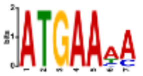	2.1e-012	199
3.	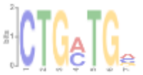	3.5e-001	149
4.	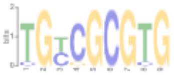	9.0e-001	19
5.	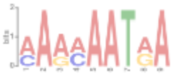	1.6e+001	63
6.	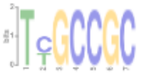	1.7e+002	45
7.	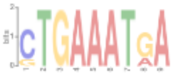	5.6e+002	16
8.	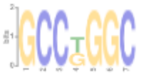	9.0e+002	39
9.	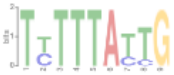	1.2e+003	25
10.	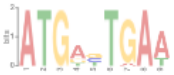	2.6e+003	49

Supplementary Table 4. Analyses of sequences motifs in RTS regions of E. coli. Logo plots of sequence motifs detected in the RTS regions across the *E. coli* genome significantly enriched sequences are only 1-2 in each column. E-value represents the probability of this motif to appear by chance, and Sites represent the number of genes that harbor this motif in the expected RTS region.

To test for the existence of conserved sequence motifs located near the stop codon, in the expected RTS region, which may account for the observed increase in folding energy, we used the MEME algorithm[5] on the relevant sequences for putative RTS sequences and non-RTS sequences from all *E. coli* genes, all sequences are within the region of -10 to +60 bases around the stop codon of each gene. (table S6, for annotation explanation, see Methods). We limited the search to motifs with a length of 3-9nt and the number of motifs to 15 (top 10 results shown below).

The putative RTS regions contain two significantly enriched motifs. First, TTTTT was found in 359/2287 of the sequences (sites), which are the known Rho-independent terminator's uridine stretch[3]. Second, ATAAAAAA, found in 148/2287 sequences. This motif is of unknown function to us. However, since it is present in a relatively small fraction of the genes, we did not further characterize it.

The putative non-RTS regions also contain two significantly enriched motifs. First, GCTGGC was found in 95/1809 sequences. This motif is of unknown function to us. However, since it is present in a relatively small fraction of the genes, we did not further characterize it. Second, ATGAA, found in 199/1809 sequences, represent a start-codon related enriched motif in downstream operon CDSs.

Supplementary data tables parameter annotations:

Supplementary data file 4: Data for all *E. coli* genes

Field	Description
Gene_LocusTag	NCBI taxonomic identifier of the gene
TaxId	NCBI taxonomic identifier of the species
3Utr_Intergenic_Length	Distance from the end of this CDS to start of downstream CDS (on either strand), 3UTR_intergenic_length_(from upstream stop codon todownstream_start-codon), distance in nucleotides
Protein abundance data	% of genes for which the downstream CDS is transcribed on the other strand (therefore cannot be found in the middle of an operon)
dLFE_in_pos_XX	ΔLFE [kcal mol^{-1} $window^{-1}$] in the XX position from the first nucleotide of the 3' UTR, i.e., the nucleotide after the stop codon of each gene. It is calculated for each position from -50 to +49.
gene_position_within_operon	Position of the gene in its operon, if this gene is in a monocistronic mRNA, or first in the operon, position will be 0.
Number_of_genes_in_operon	No further annotation is needed
gene_DNA_strand	The strand (either + or -) of the gene
next_gene_DNA_strand	The strand (either + or -) of the neighboring downstream gene

Supplementary data files 5a-c: Data averages for all 128 bacterial species analyzed:

Field	Description
TaxId	NCBI taxonomic identifier of the species
3UtrLength	The average distance from the end of this CDS to start of downstream CDS (on either strand)
NextOpposite	% of genes for which the downstream CDS is transcribed on the other strand (therefore cannot be found in the middle of an operon)
GC	Genomic %GC
RTS	Mean peak folding strength found at the RTS region (-10nt to +20nt relative to 3'-UTR start), in units of kcal mol^{-1} $window^{-1}$.
RTSfrac	% of genes matching the RTS definition (see above).
GenomeSequence	Ensembl sequence for this genome
GenomeAnnot	Ensembl annotation for this genome, in gff3 format

Supplementary data files 5b and 5c:

Field	Description
TaxId	NCBI taxonomic identifier of the species
-100, -99, -90, … 98	Mean LFE values (Native LFE or ΔLFE), for the species matching TaxId, for the window starting at position -100nt relative to the first nucleotide in the 3'-UTR, in units of kcal mol^{-1} $window^{-1}$.<br>E.g., position -3 is the window starting at the first nucleotide of the start codon.

Supplementary data file 6: Data averages for all 128 bacterial species analyzed:

Field	Description
ProtId	Ensembl identifier for this gene
RTSfound	Does the region around the stop codon match the RTS definition (see below).
RTSFromPosRelative	Position of the start of the RTS region, in nucleotides, relative to the position of the first nucleotide of the 3'-UTR. (or 0 if RTSfound=False)
RTSToPosRelative	Position of the end of the RTS region, in nucleotides, relative to the position of the first nucleotide of the 3'-UTR. (or 0 if RTSfound=False)
RTSFromPosGenomic	Position of the start of the RTS region, in nucleotides, relative to the chromosome or contig specified in ChromosomeOrContig. (or, if RTSfound=False, the position 10nt upstream of the 3'-UTR)
RTSToPosGenomic	Position of the end of the RTS region, in nucleotides, relative to the chromosome or contig specified in ChromosomeOrContig. (or, if RTSfound=False, the position 20nt downstream of the start of the 3'-UTR)
Strand	Transcription strand for this gene
NextCDSonOppositeStrand	Are the downstream CDS transcribed from the opposite strand (True) or from the same strand (False)
ChromosomeOrContig	Name of chromosome or contig from which the RTS sequence can be found (using the position specified by RTSFromPosGenomic, RTSFromPosGenomic, Strand)
TaxId	NCBI taxonomic identifier of the species

Synthetic Operon Sequence:

The RFP stop codon is colored in red, followed by the fixed 6-nucleotides (purple) and the 24-nucleotides random sequence, which vary between clones (green).

ATGGCTTCCTCCGAAGACGTTATCAAAGAGTTCATGCGTTTCAAAGTTCGTATGGAAGGTTC
CGTTAACGGTCACGAGTTCGAAATCGAAGGTGAAGGTGAAGGTCGTCCGTACGAAGGTACC
CAGACCGCTAAACTGAAAGTTACCAAAGGTGGTCCGCTGCCGTTCGCTTGGGACATCCTGTC
CCCGCAGTTCCAGTACGGTTCCAAAGCTTACGTTAAACACCCGGCTGACATCCCGGACTACC
TGAAACTGTCCTTCCCGGAAGGTTTCAAATGGGAACGTGTTATGAACTTCGAAGACGGTGGT
GTTGTTACCGTTACCCAGGACTCCTCCCTGCAAGACGGTGAGTTCATCTACAAAGTTAAACT
GCGTGGTACCAACTTCCCGTCCGACGGTCCGGTTATGCAGAAAAAAACCATGGGTTGGGAA
GCTTCCACCGAACGTATGTACCCGGAAGACGGTGCTCTGAAAGGTGAAATCAAAATGCGTCT
GAAACTGAAAGACGGTGGTCACTACGACGCTGAAGTTAAAACCACCTACATGGCTAAAAA
CCGGTTCAGCTGCCGGGTGCTTACAAAACCGACATCAAACTGGACATCACCTCCCACAACGA
AGACTACACCATCGTTGAACAGTACGAACGTGCTGAAGGTCGTCACTCCACCGGTGCTATGG
GATCCGCTGGCTCCGCTGCTGGTTCTGGCGAA**TAGACTAGTNNNNNNNNNNNNNNNNNN**
NNNNNAAGGGCGAGGAGCTCTTTACTGGCGTAGTACCAATTCTCGTAGAGCTCGATGGCGA
TGTAAATGGCCATAAGTTTTCCGTACGCGGCGAGGGCGAGGGCGATGCAACTAACGGCAAG
CTCACTCTCAAGTTTATTTGTACTACTGGCAAGCTCCCAGTACCATGGCCAACTCTCGTAACT
ACTCTGACCTATGGCGTACAATGTTTTTCCCGCTATCCAGATCACATGAAGCAACATGATTTT
TTTAAGTCCGCAATGCCAGAGGGCTATGTACAAGAGCGCACTATTAGCTTTAAGGATGATGG
CACCTATAAGACTCGCGCAGAGGTAAAGTTTGAGGGCGATACTCTCGTAAATCGCATTGAGC
TCAAGGGCATTGATTTTAAGGAGGATGGCAATATTCTCGGCCATAAGCTGGAGTATAATTTC
AATTCCCATAATGTATATATTACCGCAGATAAGCAAAAGAATGGCATTAAGGCGAATTTTAA
GATTCGCCATAATGTGGAGGATGGCTCCGTACAACTCGCAGATCATTATCAACAAAATACTC
CAATTGGCGATGGCCCAGTACTCCTCCCAGATAATCATTATCTCTCCACTCAATCCGTGCTCT
CCAAAGATCCAAATGAGAAGCGCGATCACATGGTACTCCTGGAGTTTGTAACTGCAGCAGG
CATTACTCATGGCATGGATGAGCTCTATAAGCTCGAGCACCACCACCACCACCACTAA

Monocistronic GFP Sequence (ΔRFP):

The Lac operator is colored in orange, the 18 bases from the RFP gene that were left-in are colored in red, followed by the fixed 6-nucleotides (purple) and the 24-nucleotides random sequence, which vary between clones (green).

T**TGCTTTGTGAGCGGATAACAATTATAATAGATTCAATTGTGAGCGGATAACAATTTCA**
CCACAGAAACAGAA**GCTGGTTCTGGCGAATAGACTAGTNNNNNNNNNNNNNNNNNNNNN**
NNNAAGGGCGAGGAGCTCTTTACTGGCGTAGTACCAATTCTCGTAGAGCTCGATGGCGATGT
AAATGGCCATAAGTTTTCCGTACGCGGCGAGGGCGAGGGCGATGCAACTAACGGCAAGCTC
ACTCTCAAGTTTATTTGTACTACTGGCAAGCTCCCAGTACCATGGCCAACTCTCGTAACTACT
CTGACCTATGGCGTACAATGTTTTTCCCGCTATCCAGATCACATGAAGCAACATGATTTTTTT
AAGTCCGCAATGCCAGAGGGCTATGTACAAGAGCGCACTATTAGCTTTAAGGATGATGGCA
CCTATAAGACTCGCGCAGAGGTAAAGTTTGAGGGCGATACTCTCGTAAATCGCATTGAGCTC
AAGGGCATTGATTTTAAGGAGGATGGCAATATTCTCGGCCATAAGCTGGAGTATAATTTCAA

TTCCCATAATGTATATATTACCGCAGATAAGCAAAAGAATGGCATTAAGGCGAATTTTAAGA
TTCGCCATAATGTGGAGGATGGCTCCGTACAACTCGCAGATCATTATCAACAAAATACTCCA
ATTGGCGATGGCCCAGTACTCCTCCCAGATAATCATTATCTCTCCACTCAATCCGTGCTCTCC
AAAGATCCAAATGAGAAGCGCGATCACATGGTACTCCTGGAGTTTGTAACTGCAGCAGGCA
TTACTCATGGCATGGATGAGCTCTATAAGCTCGAGCACCACCACCACCACCACTAA

Supplementary References

1. Hecht, A. *et al.* Measurements of translation initiation from all 64 codons in E. coli. *Nucleic Acids Res.* **45**, 3615–3626 (2017).
2. Espah Borujeni, A. & Salis, H. M. Translation Initiation is Controlled by RNA Folding Kinetics via a Ribosome Drafting Mechanism. *J. Am. Chem. Soc.* **138**, 7016–7023 (2016).
3. Mitra, A., Kesarwani, A. K., Pal, D. & Nagaraja, V. WebGeSTer DB-A transcription terminator database. *Nucleic Acids Res.* **39**, 129–135 (2011).
4. Gama-Castro, S. *et al.* RegulonDB version 9.0: High-level integration of gene regulation, coexpression, motif clustering and beyond. *Nucleic Acids Res.* **44**, D133–D143 (2016).
5. Bailey T. L., & Elkan C. Fitting a mixture model by expectation maximization to discover motifs in biopolymers, Proceedings of the Second International Conference on Intelligent Systems for Molecular Biology, pp. 28-36, AAAI Press, Menlo Park, California, 1994.
