# Supplementary material for: A possible universal role for mRNA secondary structure in bacterial translation revealed using a synthetic operon
Source: Nat Commun. 2020 Sep 24;11:4827. doi: 10.1038/s41467-020-18577-4 (PMC7518266; doi:10.1038/s41467-020-18577-4)
Supplement: Supplementary file 5 — Data set 3 [file 41467_2020_18577_MOESM5_ESM.pdf]

Clone	ΔG_{fold} [kcal/mol/ window]	mRNA secondary structure*
29	-8.9	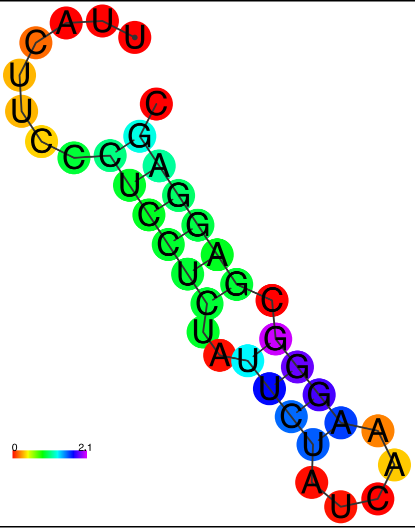
33	-9.4	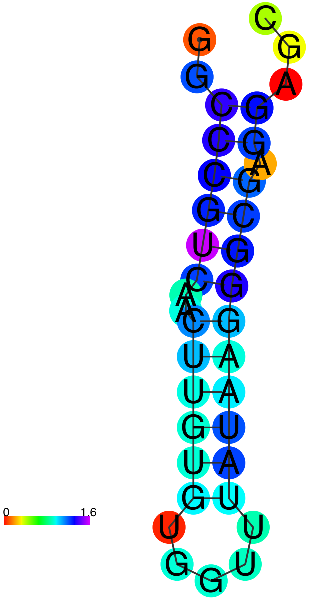
52	-1.3	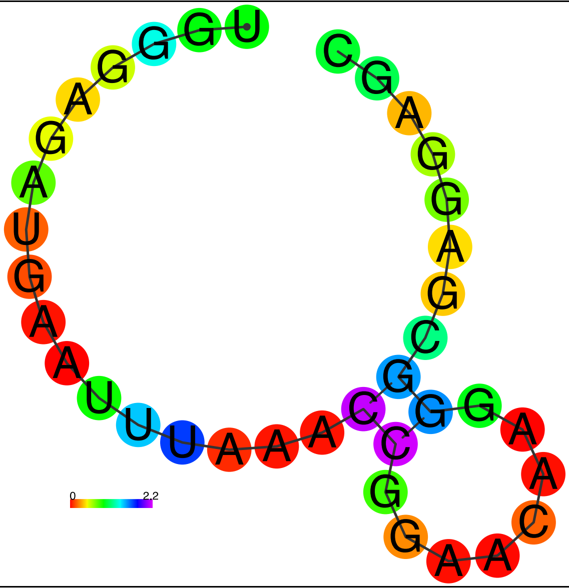

56	-8.5	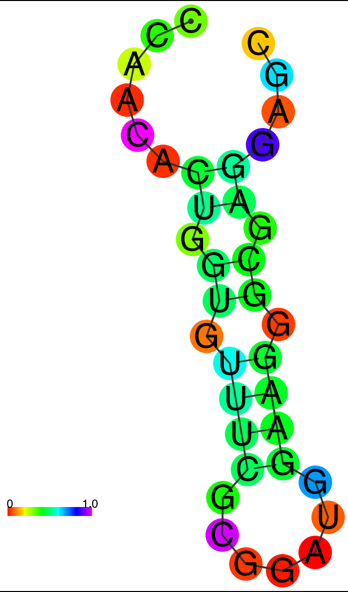
57	-6.5	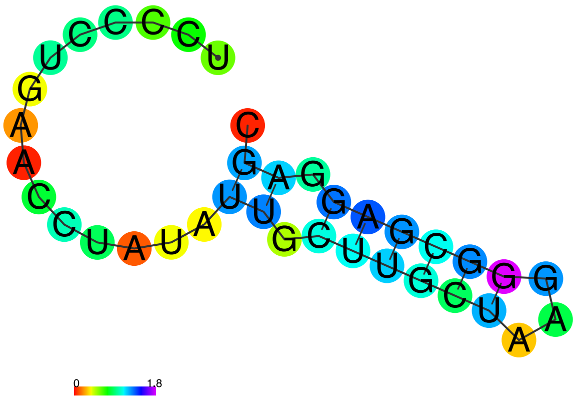
62	-6.7	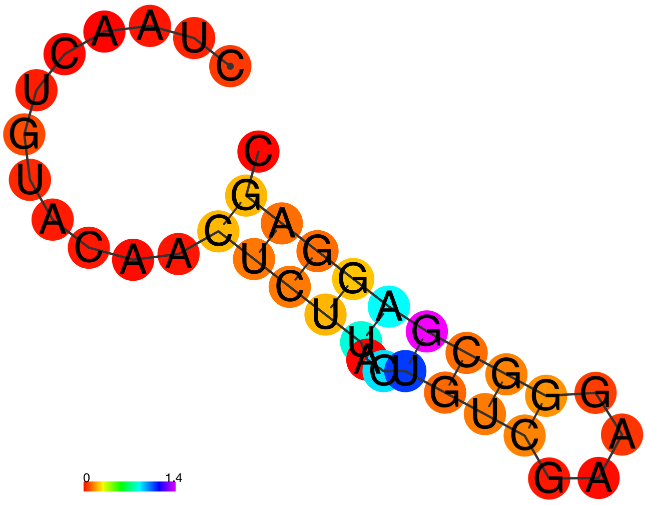

71	-4.9	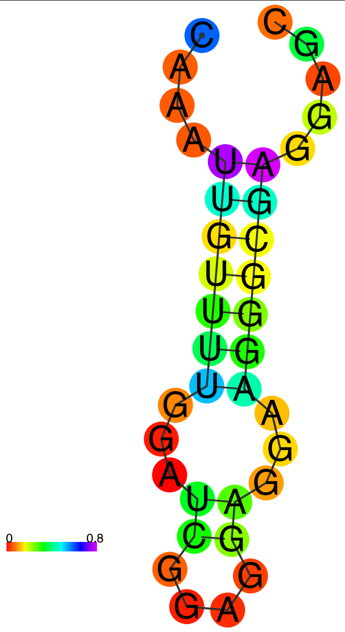
91	-2.5	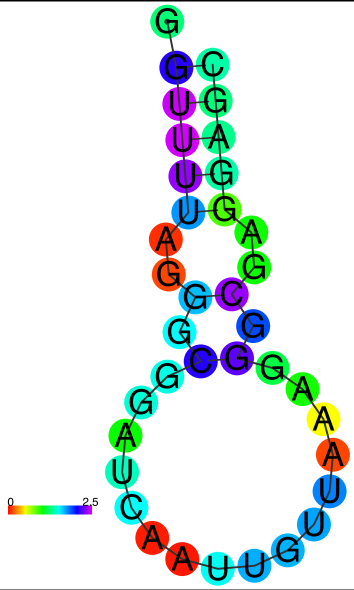
96	-2.1	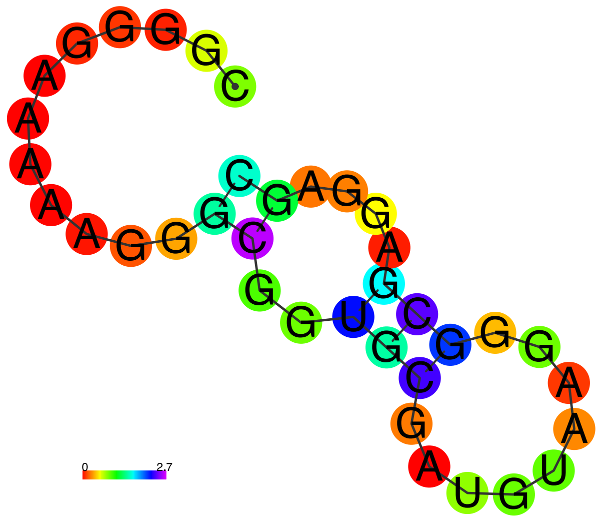

101	-6.0	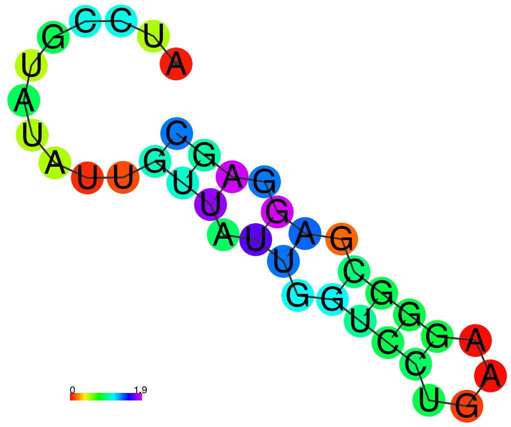
110	-8.2	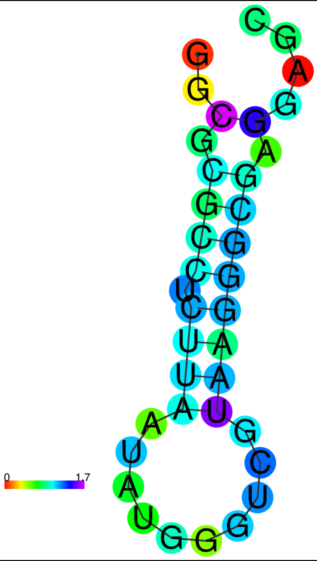
111	-13.7	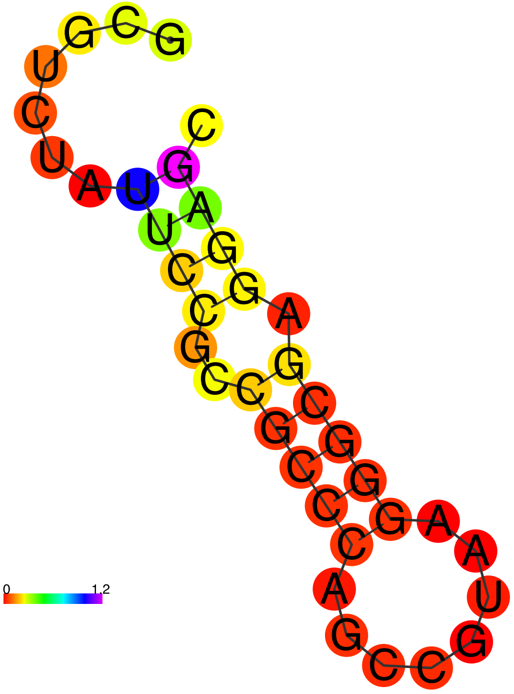

202	-11.3	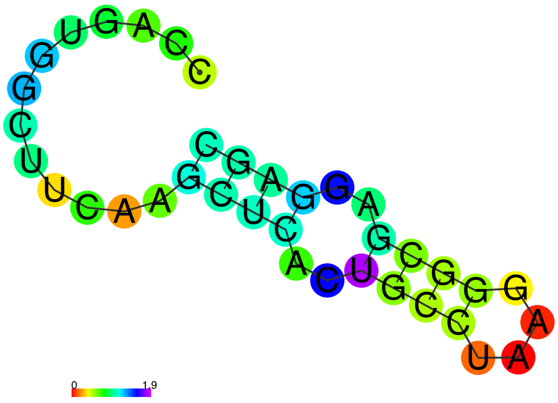
203	-7.6	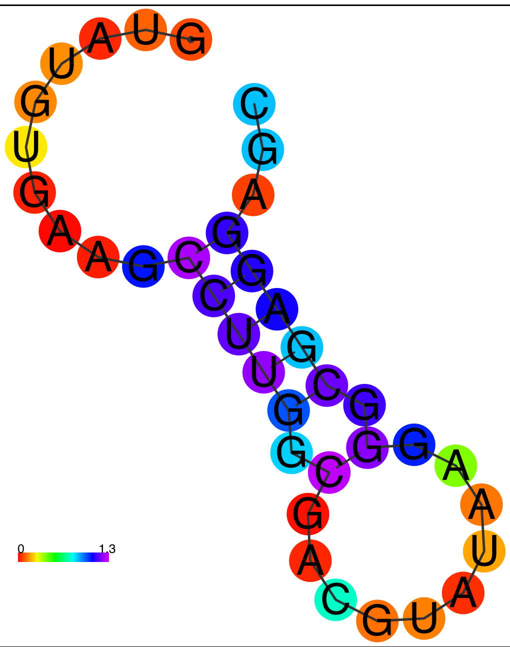
207	-1.8	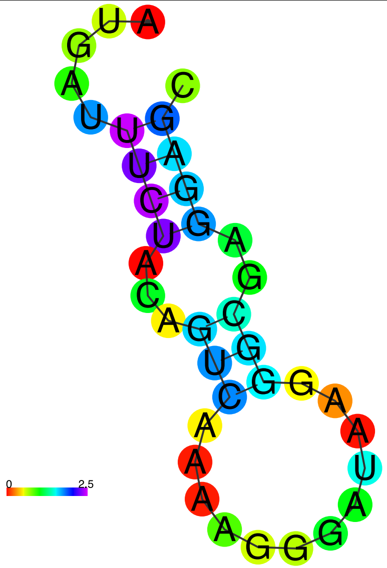

208	-1.9	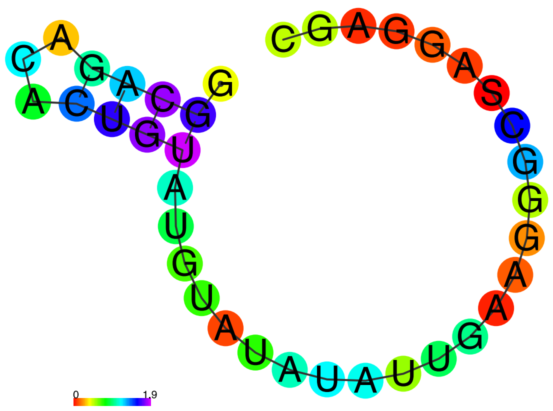
209	-4.7	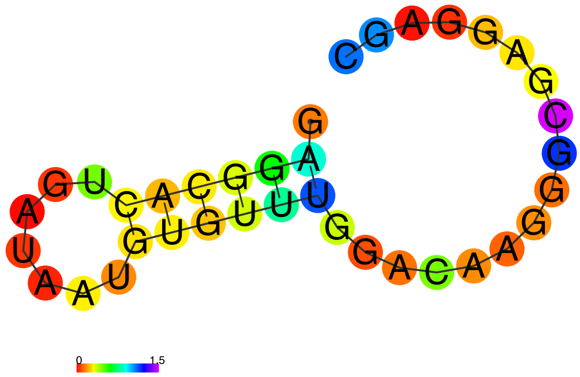
212	-5.9	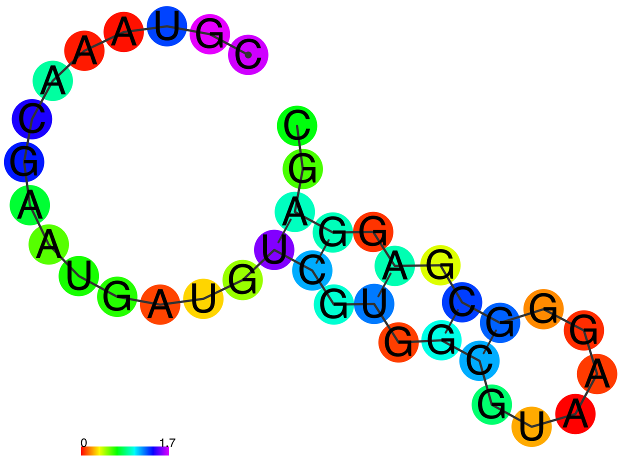

214	-2.8	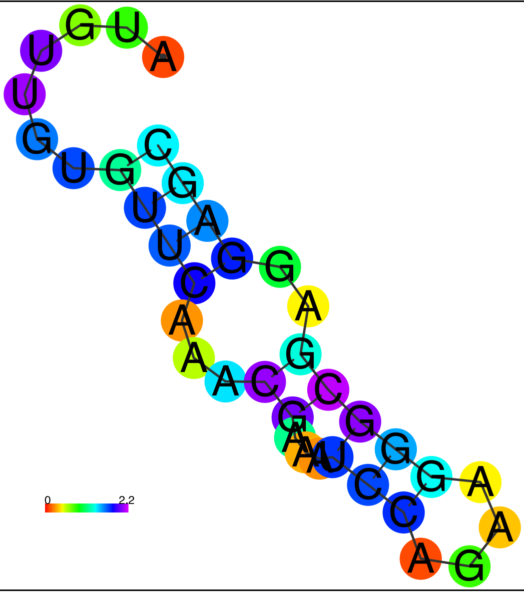
216	-0.8	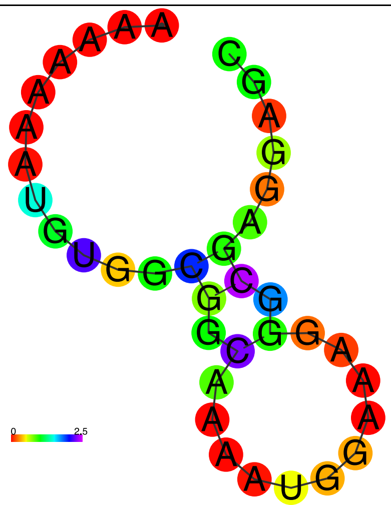
220	-3.9	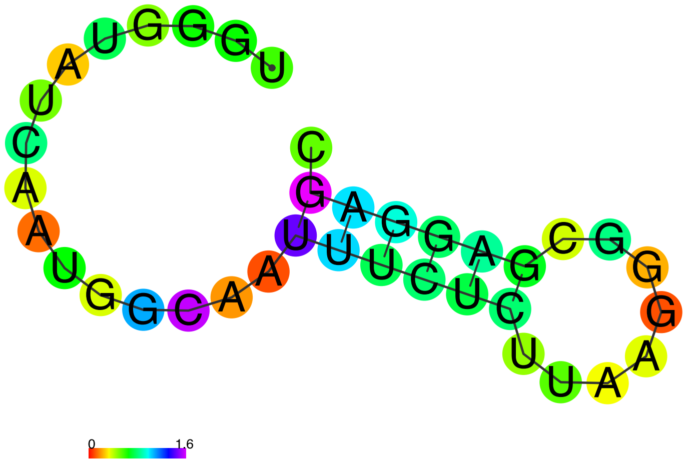

222	-6.6	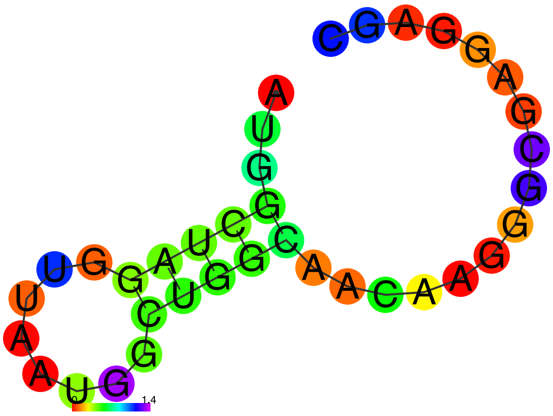
225	-7.5	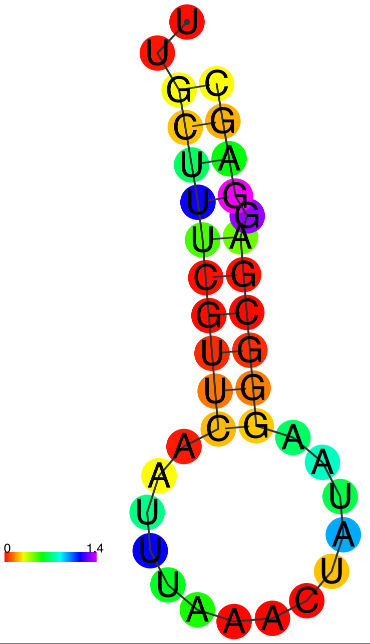
226	-9.2	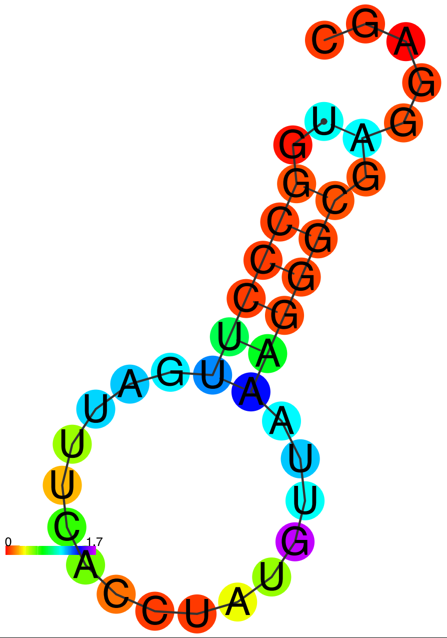

230	-5.0	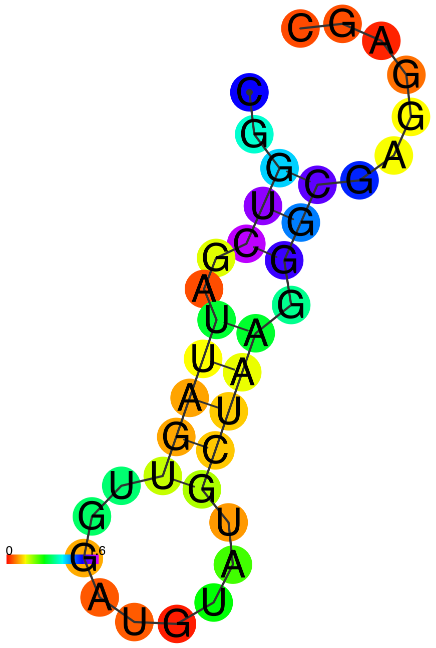
232	-7.6	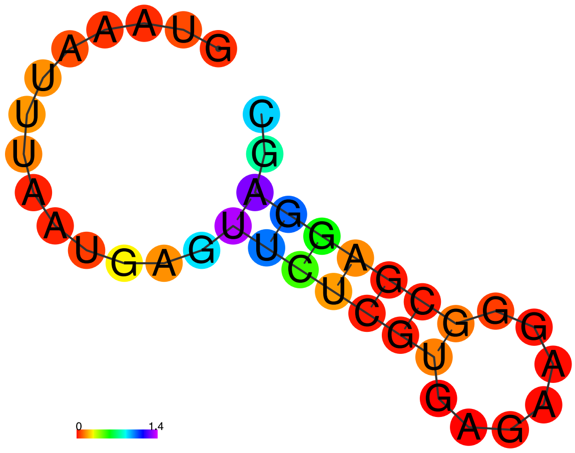
233	-8.4	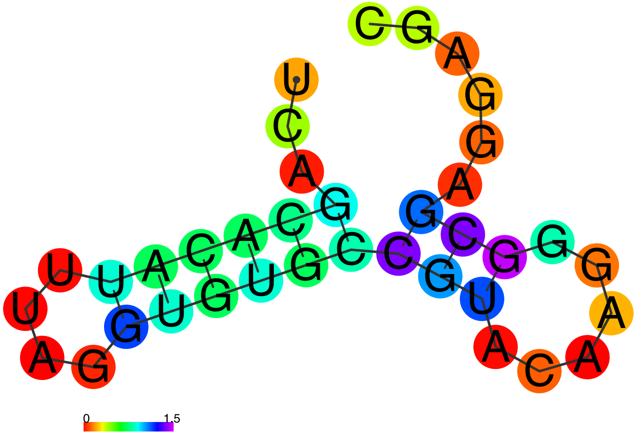

235	-9.5	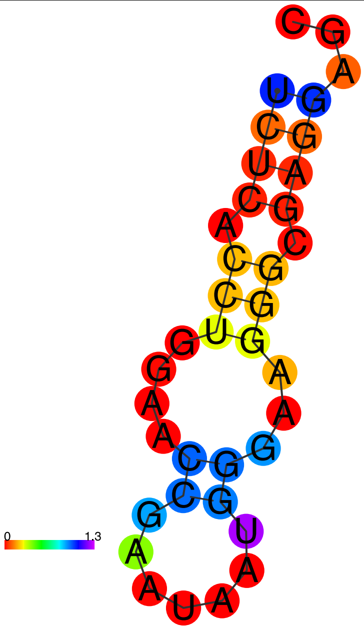
236	-7.9	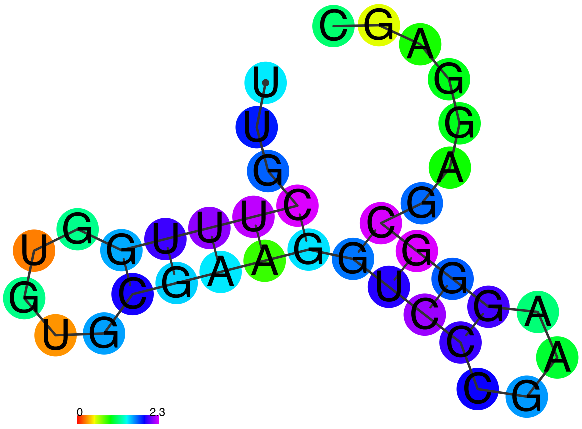
238	-8.4	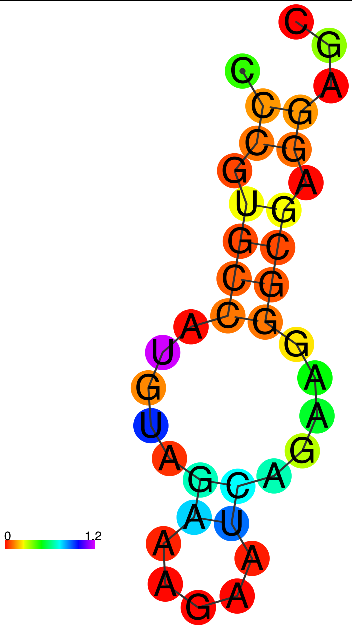

244	-3.5	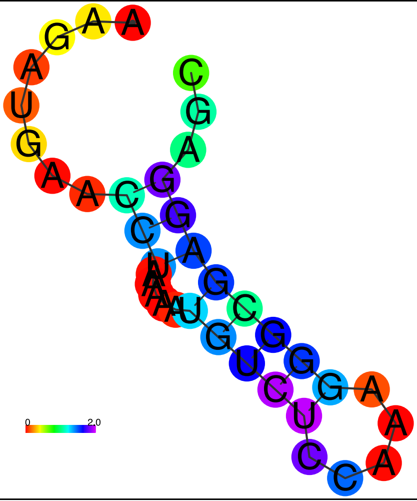
245	-5.1	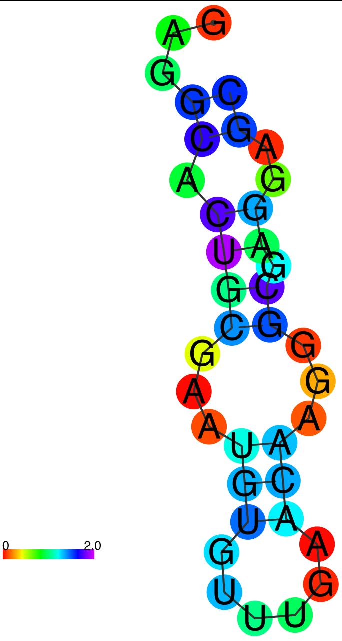
249	-1.9	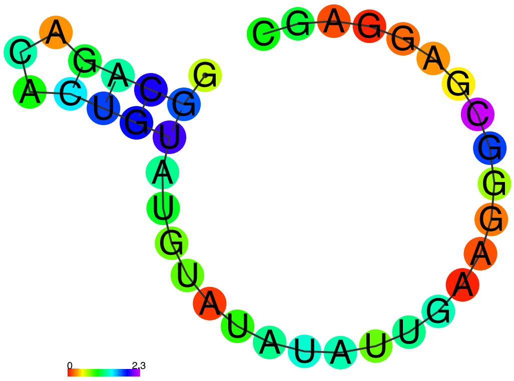

*color hue represents how well defined the structure is through a positional entropy measurement of the mountain plot of the Vienna package. The red color is highly well defined, while violet is ill-defined.
