# Supplementary material for: A possible universal role for mRNA secondary structure in bacterial translation revealed using a synthetic operon
Source: Nat Commun. 2020 Sep 24;11:4827. doi: 10.1038/s41467-020-18577-4 (PMC7518266; doi:10.1038/s41467-020-18577-4)
Supplement: Supplementary file 13 — Reporting Summary [file 41467_2020_18577_MOESM13_ESM.pdf]

nature research

Corresponding author(s): Tamir Tuller and Lital Alfonta

Last updated by author(s): Aug 24, 2020

Reporting Summary

Nature Research wishes to improve the reproducibility of the work that we publish. This form provides structure for consistency and transparency in reporting. For further information on Nature Research policies, see our Editorial Policies and the Editorial Policy Checklist.

Statistics

For all statistical analyses, confirm that the following items are present in the figure legend, table legend, main text, or Methods section.

n/a	Confirmed	
[ ]	[x]	The exact sample size (*n*) for each experimental group/condition, given as a discrete number and unit of measurement
[ ]	[x]	A statement on whether measurements were taken from distinct samples or whether the same sample was measured repeatedly
[ ]	[x]	The statistical test(s) used AND whether they are one- or two-sided<br>*Only common tests should be described solely by name; describe more complex techniques in the Methods section.*
[x]	[ ]	A description of all covariates tested
[x]	[ ]	A description of any assumptions or corrections, such as tests of normality and adjustment for multiple comparisons
[ ]	[x]	A full description of the statistical parameters including central tendency (e.g. means) or other basic estimates (e.g. regression coefficient) AND variation (e.g. standard deviation) or associated estimates of uncertainty (e.g. confidence intervals)
[ ]	[x]	For null hypothesis testing, the test statistic (e.g. *F*, *t*, *r*) with confidence intervals, effect sizes, degrees of freedom and *P* value noted<br>*Give P values as exact values whenever suitable.*
[x]	[ ]	For Bayesian analysis, information on the choice of priors and Markov chain Monte Carlo settings
[x]	[ ]	For hierarchical and complex designs, identification of the appropriate level for tests and full reporting of outcomes
[x]	[ ]	Estimates of effect sizes (e.g. Cohen's *d*, Pearson's *r*), indicating how they were calculated

Our web collection on statistics for biologists contains articles on many of the points above.

Software and code

Policy information about availability of computer code

Data collection	Genomic, protein abundance, operon data was collected directly from public databases. Next generation sequenceing data was collected using the Miseq (Illumina, USA) software. FACS data was collected using FACSAria II, BD Biosciences software. Electrophoresis densitometry data was collected using an Image J software V1.52a, Bio-Rad CFX Manager V3.1 software,FlowJo version V 10.6.1. all details were added to the methods section.
Data analysis	R package ggplot (version 3.2.1). R package date2 (version 1.14). ViennaRNA (version 2.4.9) Python (version 3.7.3) . Numpy (version 1.18.1) Scikit (version 1.3.2), Biopython (version1.74), ETE3 toolkit (version 3.1.1.). Pandas (version 0.25.3)Metplotlib (version 3.1.1). Custom codes are available on GitHub public repository: [https://github.com/michaelpeeri/rnafold-rts-public]. all details were added to the methods section.

For manuscripts utilizing custom algorithms or software that are central to the research but not yet described in published literature, software must be made available to editors and reviewers. We strongly encourage code deposition in a community repository (e.g. GitHub). See the Nature Research guidelines for submitting code & software for further information.

Data

Policy information about availability of data

All manuscripts must include a data availability statement. This statement should provide the following information, where applicable:

- Accession codes, unique identifiers, or web links for publicly available datasets
- A list of figures that have associated raw data
- A description of any restrictions on data availability

All data generated or analyzed during this study are included in this published article and its supplementary information and data files.

Field-specific reporting

Please select the one below that is the best fit for your research. If you are not sure, read the appropriate sections before making your selection.

☒ Life sciences ☐ Behavioural & social sciences ☐ Ecological, evolutionary & environmental sciences

For a reference copy of the document with all sections, see nature.com/documents/nr-reporting-summary-flat.pdf

Life sciences study design

All studies must disclose on these points even when the disclosure is negative.

Sample size	All sample sizes were chosen under the constraints of the experimental systems to allow enough statistical power. For example, the sample size of FACS-NGS experiment was limited by the transformation efficiency and competence of a random library of clones into bacteria. However, the sample size was predicted and found that it allows enough statistical power. Computational analysis sample sizes include all available data.
Data exclusions	The only experimental data that was excluded, are of synthetic operon which did not have efficient start codons and /or harboured additional stop codons in the variable region. AS described in the methods section.
Replication	All experiments were replicated more than once, all replications were successful and within the errors of the measurements.
Randomization	1) Randomized sequences (in the mRNA stability analysis) were sampled from the distribution representing the null hypothesis, namely, only the amino acids sequence, nucleotides and codon composition (see below) were under selection at a given position in the coding sequence. While only nucleotide composition was under selection in a given UTR. To produce random sequences maintaining these properties, synonymous codons within each coding sequence were randomly per mutated and the nucleotides of each UTR were randomly per mutated. Regions overlapping multiple coding sequences were maintained without permutations. Codons containing one or more ambiguous nucleotides ('N' bases) were likewise maintained without permutations. Synonymous codons were identified according to the gene translation table for each specie.<br>2) Randomization of the 24 nucleotides library in the recombinant construct was achieved by random synthesis of the library, without a specific nucleotide bias or constrains in the synthesis.<br>3) 128 bacterial strains that were analyzed in this study were randomly selected from all bacteria with sequenced genomes, with one constraint: it should have represented all bacterial Phyla.
Blinding	A randomized library of 24 nucleotides were sorted by FACS, according to GFP fluorescence. Sequencing of library was only conducted after sorting, and correlation of results was performed.

Reporting for specific materials, systems and methods

We require information from authors about some types of materials, experimental systems and methods used in many studies. Here, indicate whether each material, system or method listed is relevant to your study. If you are not sure if a list item applies to your research, read the appropriate section before selecting a response.

Materials & experimental systems

n/a	Involved in the study	
☐	☒	Antibodies
☒	☐	Eukaryotic cell lines
☒	☐	Palaeontology and archaeology
☒	☐	Animals and other organisms
☒	☐	Human research participants
☒	☐	Clinical data
☒	☐	Dual use research of concern

Methods

n/a	Involved in the study	
☒	☐	ChIP-seq
☐	☒	Flow cytometry
☒	☐	MRI-based neuroimaging

Antibodies

Antibodies used	anti his-tag-probe (H-3) antibodies, Santa Cruz Biosciences sc-8036, Lot #B2317 dilution 1:2000
Validation	Commercial validation and citations are available at: http://www.scbt.com/p/his-probe-antibody-h-3

Flow Cytometry

Plots

Confirm that:

- [x] The axis labels state the marker and fluorochrome used (e.g. CD4-FITC).
- [x] The axis scales are clearly visible. Include numbers along axes only for bottom left plot of group (a 'group' is an analysis of identical markers).
- [x] All plots are contour plots with outliers or pseudocolor plots.
- [x] A numerical value for number of cells or percentage (with statistics) is provided.

Methodology

Sample preparation	The source of the E. coli MG1655 strain was Yale stock CGSC#:6300. The entire population of Cells were grown and induced using IPTG, overnight to express GFP and RFP. Cells were then washed with PBS and directly subjected to Sorting.
Instrument	Flow Cytometry was performed using: FACSAria III, BD Biosciences.
Software	Analysis was performed using FlowJo software version number 10.6.1
Cell population abundance	Population abundance and statistics are detailed in figure S1 of the manuscript; target population consisted 49.7% of the entire bacterial population.
Gating strategy	The gating strategy was as follows: The preliminary FSC-A/SSC-A gates were 630-17,000 and 60-3,000, respectively, the SSC-W/SSC-H gates were 0-110,000 and 450-45,000, respectively, and the FSC-W/FSC-H gates were 12,000-62,000 and 200-4,000, respectively. Cells that expressed RFP, which served as the positive and normalizing control with levels between 3,500-15,000, were further gated. Next, the resulting population (49.7% of the total population) was gated into 8 ~equal groups divided and defined by GFP expression. Each group was intended to represent ~12.5% of the parent population.

- [x] Tick this box to confirm that a figure exemplifying the gating strategy is provided in the Supplementary Information.
